# Accuracy of the Resting Energy Expenditure Estimation Equations for Healthy Women

**DOI:** 10.3390/nu13020345

**Published:** 2021-01-24

**Authors:** Rafael Molina-Luque, Fernanda Carrasco-Marín, Constanza Márquez-Urrizola, Natalia Ulloa, Manuel Romero-Saldaña, Guillermo Molina-Recio

**Affiliations:** 1 Grupo Asociado de Investigación Estilos de Vida, Innovación y Salud, Instituto Maimónides de Investigación Biomédica de Córdoba (IMIBIC), 14004 Córdoba, Spain; rafael.moluq@gmail.com (R.M.-L.); manuelromerosal@gmail.com (M.R.-S.); en1moreg@uco.es (G.M.-R.); 2 Departamento de Enfermería, Farmacología y Fisioterapia, Facultad de Medicina y Enfermería, Universidad de Córdoba, 14004 Córdoba, Spain; 3 Centro de Vida Saludable y Departamento de Bioquímica Clínica e Inmunología, Facultad de Farmacia, Universidad de Concepción, 4070386 Concepción, Chile; contymarquez@gmail.com (C.M.-U.); nulloa@udec.cl (N.U.)

**Keywords:** anthropometry, resting energy expenditure, women

## Abstract

Background: There exist several prediction equations for the estimation of resting energy expenditure (REE). However, none of these equations have been validated in the Chilean female population yet. The aims of this study are (1) to determine the accuracy of existing equations for prediction of REE and (2) to develop new equations in a sample of healthy Chilean women. Methods: A cross-sectional descriptive study was carried out on 620 Chilean women. The sample showed an age range between 18 and 73 years, a body mass index average of 28.5 ± 5.2 kg/m^2^, and a prevalence of overweight and obesity of 41% and 33.2%, respectively. REE was measured by indirect calorimetry (REE_IC_), which was used as the gold standard to determine the accuracy of twelve available REE prediction equations and to calculate alternative formulas for estimation of REE. Paired t-tests and Bland–Altman plots were used to know the accuracy of the estimation equations with REE_IC_. At the same time, multiple linear regressions were performed to propose possible alternative equations. The analyses were carried out by age groups and according to nutritional status. Results: All the equations showed a tendency to overestimate REE, regardless of age or nutritional status. Overall, the Ireton-Jones equation achieved the highest mean percentage difference from REE_IC_ at 67.1 ± 31%. The alternative new equations, containing variables of body composition, reached a higher percentage of classification within ±10% of REE_IC_. Conclusions: The available equations do not adequately estimate REE in this sample of Chilean women. Although they must be validated, the new formulas proposed show better adaptation to this Chilean sample.

## 1. Introduction

Mortality due to non-communicable diseases (NCDs) has been increasing steadily in recent years. Currently, NCDs cause 41 million deaths per year, representing 71% of the total number of deaths worldwide [1]. These include those due to cardiovascular diseases (the world’s leading cause of death), cancer, respiratory diseases, and diabetes [2]. NCDs may increase mortality rates and decrease life quality by reducing disability-adjusted life years, life expectancy, and potential life years lost [3].

NCDs’ development is related to several cardiovascular risk factors (CRF) [4,5,6]. Among the CFR, overweight and obesity stand out. These conditions are considered as severe public health problems because of their high prevalence and impact on health at all life stages [7,8]. It should be noted that this problem is more prevalent in the female than in the male population. In 2016, the prevalence of overweight was estimated at 39% in men and 40% in women, and the prevalence of obesity reached 11% in men and 15% in women [9]. For instance, between 1975 and 2016, worldwide, women suffering from obesity increased from 69 to 390 million, while in men, this rate varied from 31 to 281 million [10]. This problem also impacts the economic and social aspects of families and national health systems [11].

For these reasons, early lifestyle interventions are essential to ensure good health. It is necessary to emphasize that diet plays a fundamental role in the development of NCDs [12]. Although improving food quality and controlling the percentages of macronutrients that provide daily energy is crucial, an essential element of any dietary approach aimed at body fat loss is the relationship between energy intake and expenditure (energy balance) [13]. A positive energy balance (energy intake > energy expenditure) leads to weight gain, so all recommendations on body fat loss include energy intake reduction and an increase in energy expenditure, primarily through physical activity, to ensure a proper energy flux [14,15]. To know the total energy expenditure (TEE) is fundamental to be able to adjust this relationship. TEE is composed of the thermic effect of activity, non-exercise activity thermogenesis, the thermic effect of food, and resting energy expenditure (REE) [15]. Of these four TEE components, REE represents the highest proportion, reaching between 60 and 70%, depending on the level of physical activity performed [16]. Therefore, to adjust the energy intake, it is necessary to estimate REE, for which there are various methods available such as direct and indirect calorimetry. However, access to the devices that allow their measurement is not easy because they are sophisticated equipment that requires specially trained staff and entails a high cost [17].

Alternatively, there are different prediction equations available that allow estimation of REE based on more accessible parameters (e.g., age, height, weight). In this sense, sex is an essential variable in estimating REE, and it is used in the most common predictive equations because women generally have a lower REE than men [18]. These equations allow for quicker and low-cost utilization without requiring trained personnel [19]. However, these formulas are not adapted to all populations, which leads to a lack of accuracy when their results are compared with data collected using a gold standard (e.g., indirect calorimetry) [20]. In addition to the lack of accuracy, the wide variety of equations available makes it difficult to choose the most appropriate one.

Several studies have reported that these discrepancies may be increased depending on the characteristics of the study population (origin, altered metabolic state, pathologies) [21]. Two factors with a significant influence on the accuracy of the equations are age and body weight [22]. Considering age is very important because REE decreases as aging progresses [23]. In this sense, and given the significant variability of formulas (according to individuals’ age group and nutritional status) for the REE estimate [22,24], it is very complex to extrapolate and standardize the results to allow comparisons between different populations. For this reason, researchers recommend using these formulas in populations with similar characteristics to those from which they were initially developed. If they are utilized in other populations with different characteristics, it is recommended to validate or adapt them to know or improve their accuracy [22,23,24,25,26].

Therefore, this study aims (1) to determine the accuracy of twelve already available equations for estimation of REE and (2) to develop new, more accurate equations in a sample of healthy Chilean women.

## 2. Materials and Methods

### 2.1. Design, Population, and Sample

A cross-sectional descriptive study was carried out, including women who attended the nutrition consultation at the Healthy Life Centre (Concepción, Chile) between January 2016 and June 2019, where indirect calorimetry was performed. The sample size was estimated through Epidat 4.2. (Department of Health, Xunta de Galicia, Galicia, Spain). For the sample size calculation, the female population over 18 years of age in Chile was taken as a reference. The minimum sample size calculated was 537 subjects for a precision of 4%, a power of 80%, a confidence level of 95% (α = 0.05), and an expected overweight prevalence of 33.7% [27].

Women over 18 years of age were included, and those with chronic pathologies that could affect REE (such as cancer, hyperthyroidism, or hypothyroidism) or who presented an error on indirect calorimetry (given by a coefficient of variation (CV, applied to the mean of VO_2_ and VCO_2_) over 10% or measurement time below 25 min) were excluded. From the initial sample of 653 women, 33 were dismissed.

### 2.2. Antropometrics Measurements

The independent variables collected were age (years) and anthropometric measurements: weight (kg), height (cm), body mass index (BMI, kg/m^2^), body fat percentage (BF%), fat mass (FM, kg), fat-free mass (FFM, kg), and body water (BW, kg).

The anthropometric measurements were collected following the recommendations of the manual of standardized anthropometry [28]. Since the calorimeter demands anthropometric data (weight, height, and lean mass), the measurement was made before the indirect calorimetry study. For measuring height, a SECA 700 (Seca GmbH, Hambrug, Germany) stadiometer and balance were used, with an accuracy of 0.1 cm. The height measurement was made without shoes, with the feet together, heels, buttocks, and upper back touching the stadiometer, with the head in the plane of Frankfort, after a deep inspiration. We utilized the Tanita BC-418 (TANITA, Tokyo, Japan) with eight electrodes in the study of body composition. Body composition and weight were measured with light clothing and bare feet, removing metal objects such as earrings, watches, and bracelets. In addition, fasting (10–12 h), empty bladder, abstinence from alcohol or stimulant drinks, and no physical exercise 24 h before the day of the study were required for measurement. All the measures were taken by specialized personnel. Each measurement was repeated three times by the same assessor, and the average of the three value was calculated and used for further analysis.

The nutritional status classification was made according to the BMI following the WHO’s recommendations: normal weight, 18.5 to <25 kg/m^2^; overweight, 25 to <30 kg/m^2^; obesity, ≥30 kg/m^2^ [29].

### 2.3. Indirect Calorimetry and Estimation Formulas

REE (result variable) was measured using indirect calorimetry (gold standard). The modified Weir formula was used for its calculation, based on the oxygen consumed and the carbon dioxide produced [30].

REE (Kcal/day) = [(VO_2_ × 3.941) + [(VCO_2_ × 1.11)] × 1440

REE was measured using the Ultima CCM device (MGC Diagnostics, Minnesota, USA), designed to measure indirect calorimetry through respiratory gases. Before the measurement, the flow and equipment were calibrated, controlling the environmental temperature (21.6 ± 0.7 °C), the humidity (48.3 ± 5.4%), and the room’s atmospheric pressure. The measurements were made at 12 m above sea level. The REE measurement was made during the morning between 8:00 and 10:00 a.m. All women who underwent indirect calorimetry followed a strict protocol in which they were instructed to do the following: fast for 12 h, not to consume stimulant beverages, not to smoke, and not to perform physical activity in the 12 h before the test. Once the women arrived at the center for the assessment, they rested for 30 min. During calorimetry, participants were in the supine position with a face piece on, awake in the room. The process lasted 25 min, without considering the first five minutes of the REE measurement. The values’ average of the remaining 20 min was used to compute REE.

Further, we estimated REE (Kcal/day) through the formulas presented in Table 1.

### 2.4. Ethical and Legal Aspects

The study was carried out in compliance with the fundamental principles laid down in the Declaration of Helsinki (1964), the Council of Europe Convention on Human Rights and Biomedicine (1997), and the UNESCO Universal Declaration on the Human Genome and Human Rights (1997), as well as with the requirements laid down in Chilean legislation in the fields of biomedical research, the protection of personal data, and bioethics. The Bioethics Committee of the Vice-Rectory of Research of the University of Concepción approved the study protocol (October 2019; Code: 538-2019).

### 2.5. Statistical Analysis

The quantitative variables are presented with mean and standard deviation, and the qualitative values are shown with frequencies and percentages.

To contrast the goodness of fit to a normal distribution of the data from the quantitative variables, the Kolmogorov–Smirnov test with Lilliefors correction was applied. One-way ANOVA for three means or more and the post hoc Bonferroni test were performed for the bivariate hypothesis contrast. Paired *t*-tests were performed to compare REE_IC_ and REE estimated by the formulas. For the qualitative variables, the chi-square and Fisher exact tests were used when necessary. Pearson’s correlation coefficient (r) was applied to correlate REE_IC_ with anthropometric variables and REE_IC_ with REE estimated by the formulas. The cut-off points established to assess the strength of association were: very weak, 0 to 0.19; weak, 0.2 to 0.39; moderate, 0.4 to 0.59; strong, 0.6 to 0.79; and very strong 0.8 to 1.0 [41]. We performed Cohen’s d to study the size effect of the mean differences.

Adjusted multiple linear regressions were performed to develop various predictive formulas that would fit with indirect calorimetry measurements. These regressions were developed following the stepwise regression backward method. This method begins with an equation that includes all the explanatory variables and extracts, one by one, the variables with the highest “*p* value,” until a final model is reached with all the significant explanatory variables (*p* < 0.05). To determine the models’ goodness of fit, we analyzed the standard error, the adjusted coefficient of determination, the F statistic, the collinearity analysis (computing the variance inflation factor (VIF) and the tolerance statistic), and the residuals.

The concordance between REE_IC_ and REE estimated by the equations was analyzed using Bland–Altman plots. In Bland–Altman plots, positive values show overestimation of equations and negative values show underestimation. Based on previous studies [25], we assessed the percentage difference between REE measured by the indirect calorimeter and that estimated by the equations (|REE−REEIC|REEIC×100). In addition, we studied the percentage of women whose estimated REE was within ±10% of that measured by the gold standard. All the results are shown in the total sample, considering the nutritional status, according to the age groups traditionally stated in the literature (18 to <30 years, 30 to <60 years, and ≥60 years).

The probability of an α error below 5% (*p* < 0.05) was considered statistically significant for all the hypothesis contrasts, and the confidence interval was calculated at 95%. For the statistical analysis, IBM SPSS Statistics 22.0 software (IBM, Chicago, IL, USA) was used.

## 3. Results

### 3.1. Description of the Sample

The final sample consisted of 620 women. The women showed an age range between 18 and 73 years. Following the BMI criteria, the prevalence of overweight (≥25 kg/m^2^) and obesity (≥30 kg/m^2^) was 41% (95%CI 37.1–45%) and 33.2% (95%CI 29.5–37.1), respectively. The mean CV was 4.9 ± 1.6%.

Concerning the body composition, results show that BF% increased among the age groups, although this increase was not significant among the 18 to <30 years and 30 to <60 years groups (mean difference (MD) = 1.6; *p* = 0.073). On the other hand, FM decreased significantly among the age groups, although the decrease was not significant among the 30 to <60 years and ≥60 years groups (MD = 1.4; *p* = 0.419). All variables analyzed showed significant differences between the different nutritional states, except for: (i) age between 25 to <30 and ≥30 kg/m^2^ (MD = 0.3; *p* = 1.000), (ii) height between 18.5 to <25 and 25 to <30 kg/m^2^ (MD = 0.5; *p* = 1.000), and (iii) height between 25 to <30 and ≥30 kg/m^2^ (MD = 1.1; *p* = 0.194). Table 2 shows the characteristics of the sample by age and nutritional status according to BMI. Table S1 shows size effects of each group comparison.

### 3.2. Differences between REEs Measured by Indirect Calorimetry (REE_IC_) and Estimated by Twelve Available Formulas

Table 3 and Table 4 show the accuracy of the twelve equations in the total sample, according to the age groups and the nutritional status of the women studied. For all three comparisons (total sample, according to age group, and nutritional status), the estimation equations tend to significantly overestimate what was measured through indirect calorimetry, with percentage differences of more than 10% between REE_IC_ and REE estimated by the equations. Tables S2 and S3 show size effects of each group comparison, and Table S4 shows the size effect of the paired *t*-test.

**Figure 1:**
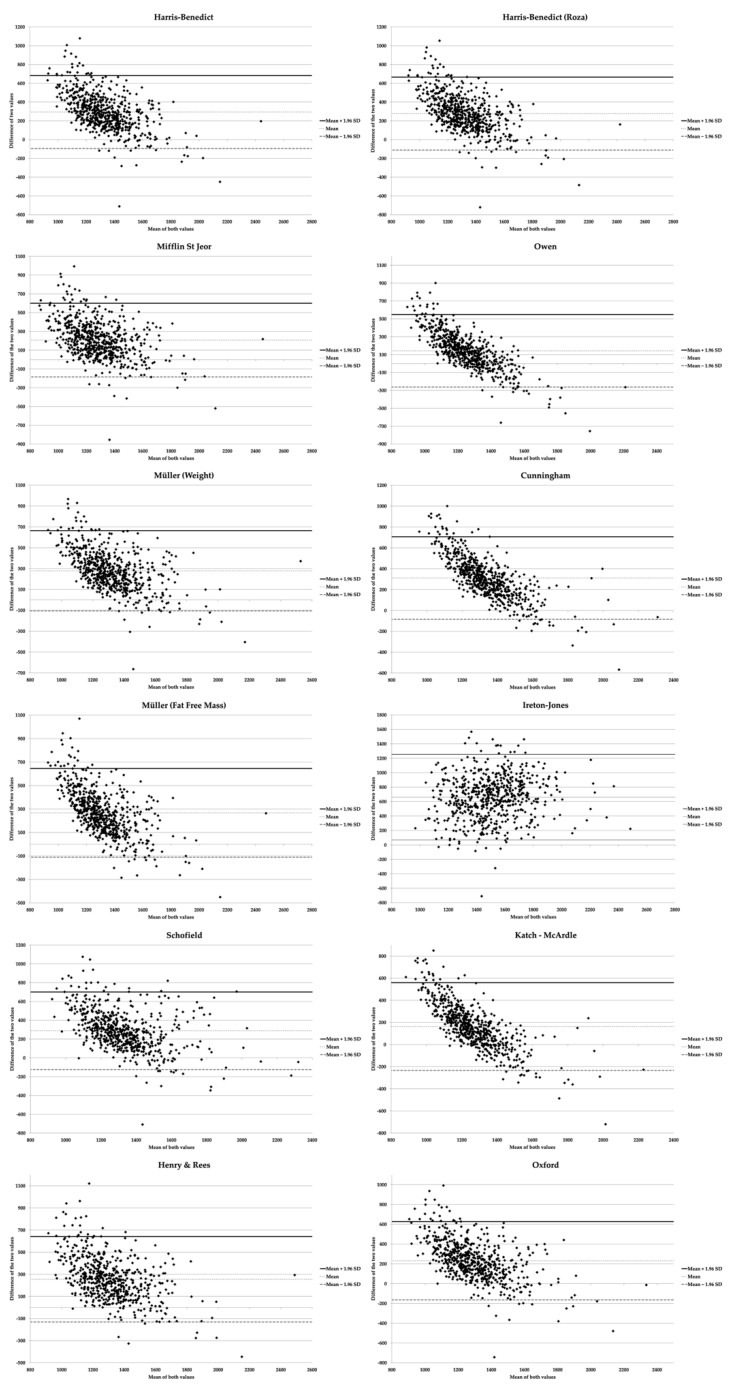
Bland–Altman graphs for resting energy expenditure equations using indirect calorimetry as the gold standard.

Figure 1 shows that, in the total sample, all the equations tended to overestimate in women with lower REE, while they reduced and even underestimated when REE increased. The equation proposed by Ireton-Jones [35] was the only one that overestimated independently of REE_IC_. Further, the mean of the differences between the calculated formulas and REE_IC_ was higher than 0 (Table 3), which confirms that, in general, the formulas tend to overestimate REE.

In the whole sample and the three age groups analyzed, the Ireton-Jones [35] equation was the one that overestimated REE the most. In contrast, the equation proposed by Owen [34] showed the lowest overall overestimate (16.5 ± 24.1%), from 18 to <30 years (13.5 ± 23.1%) and 30 to <60 years (17.1 ± 24.2%). In women ≥60 years, the Mifflin St. Jeor [32] equation showed the smallest mean difference (11.9 ± 24.2%).

Finally, the Katch-McArdle [38] equation achieved the highest proportion of classification within ±10% in the total sample (39.5%) and in the 30 to <60 age group (40.4%). In groups from 18 to <30 years and ≥60 years, the Owen [34] (43.8%) and Mifflin St. Jeor [32] (37.9%) equations showed better classification percentages, respectively. According to the BMI categories (Table 4), the Ireton-Jones [35] equation held the greatest overestimation across all nutritional status groups, and the Owen [34] equation the smallest. However, the Owen [34] equation only obtained the best classification percentage within ±10% in the group with a BMI of 18.5 to <25 kg/m^2^ (33.8%). The Katch–McArdle [38] equation was more accurate in the other two BMI groups, with 37% (25 to <30 kg/m^2^) and 53.4% (≥30 kg/m^2^). We have found discrepancies between most of the calculated equations and REEIC in women with normal weight, overweight, and ≥60 years. The correlations can explain these discrepancies because although significant, they were low (Table 3 and Table 4).

### 3.3. New Equations for the Estimation of REE

Table 5 shows the bivariate correlations between REE_IC_ and the different anthropometric variables. In the total sample, the ≥30 kg/m^2^ group and the three age groups, weight, FFM, and FM showed a moderate to strong correlation. In the normal weight and overweight groups, the correlation, although significant, was lower.

Table 6 presents several estimation formulas for the whole sample, depending on age and nutritional status. The equations that do not require the measurement of body composition showed an overestimation close to 3%, being lower in the group of 18 to <25 kg/m^2^ (2.7 ± 18.2%). The computed equations showed that about 50% of the women in the study obtained an estimation of their REE within ±10% of REE measured by indirect calorimetry. On the other hand, the formulas based on body composition data provided more homogeneity in the estimation (the 95% CI of the mean difference is closer) and higher classification percentage within ±10%, except in the group of ≥60 years and with a BMI from 18 to <25 kg/m^2^.

## 4. Discussion

The purpose of the study was to determine the accuracy of twelve already existing equations for REE estimation in a sample of healthy Chilean women. In addition, we developed several equations for this sample stratified by age and nutritional status to provide alternatives that health care professionals can use depending on their tools available (e.g., based on anthropometric or body composition values).

The prevalence of overweight and obesity among participants was 41% and 33.2%, respectively. These rates are similar to those shown by the Chilean National Health Survey (36.4% and 33.7%) [27]. All the equations analyzed tended to overestimate REE, with it being greater in those participants with lower REE. Further, while REE increases, a gradual reduction in this overestimation can be observed, reaching underestimations in the highest values. In the literature, the results between different research are not consistent. Although some authors evidenced a similar trend to that shown in our population [42,43], Anjos et al. [25] found that REE calculated in women following the Schofield [37], Henry–Rees [39], and Harris–Benedict [31] equations was higher than that measured by the indirect calorimeter, regardless of age group and BMI. Galgani et al. [44] evidenced that these equations showed a higher percentage of overestimation than underestimation. However, they achieved, globally, higher accuracy than that obtained in our results. Willis et al. [45] argued that the Mifflin St. Jeor [32] and Harris–Benedict [31] equations overestimated REE, something observed in our results, while Owen’s [34] formula underestimated it. In summary, the literature shows significant variability in the accuracy of the available formulas, mediated by various factors [46].

One of the most studied is the influence of the nutritional status, according to BMI, on REE. In this regard, the main problem found is that the authors recommend different formulas depending on the reference population, making standardization difficult. Our results show how REE_IC_ increased significantly among the normal weight (1054.4 ± 185.2 Kcal/day), overweight (1142.8 ± 198 Kcal/day), and obesity (1331.1 ± 285 Kcal/day) groups and, in general, the accuracy of the REE estimation increased with the rise in BMI. Similarly to our results, Jesus et al. [47] showed that the accuracy of the equations studied was higher among people with higher BMI. Of the analyzed equations, that proposed by Owen [34] in the normal weight group (18 to <25 kg/m^2^) and the Katch–McArdle equation [38] for the overweight (25 to <30 kg/m^2^) and obesity (≥30 kg/m^2^) groups were the ones that obtained the best classification within ±10%, with 33.8%, 37%, and 53.4%, respectively.

Although the classification improved in the overweight and obesity groups in comparison to normal weight group, it remained low due to the wide bias shown. Poli et al. [48], in their work on women suffering from obesity, reported similar results, finding a low agreement between what is predicted by the equations and what is measured by indirect calorimetry and concluding that the Harris–Benedict [31] and FAO/WHO equations offered the highest accuracy. Namazami et al. [49] recommended using the Mifflin St. Jeor [32] equation in women with normal weight and overweight (especially in overweight, where this formula has shown greater accuracy). Finally, Amaro-Gahete et al. [22,24], in their research focused on young and middle-aged adults, concluded that it was necessary to choose a different formula for each nutritional status.

Another variable that influences REE, and which a large part of the equations analyzed considers, is age. Our findings show that predictive capacity also varies depending on the age group studied. In this regard, the Owen [34], Katch–McArdle [38], and Mifflin St. Jeor [32] equations achieved a higher proportion of correct classification in the 18 to <30 years, 30 to <60 years, and ≥60 years groups, respectively. However, as with BMI, previous studies reported significant heterogeneity in recommending formulas based on population age [22,24,25].

These facts reveal that it is very complicated to establish which equation should be used in each clinical setting. This difficulty is linked to significant inter-population variability, which can be influenced by aspects such as the geographical origin [50]. In this sense, the equations analyzed were developed in samples with specific characteristics, which means that their predictive capacity decreases when testing their validity in other places. Various research has shown that REE varies according to ethnic group, mainly due to physical characteristics (abdominal fat, percentage of body fat, fat-free mass, etc.) [51,52,53]. For instance, Spaeth et al. [54] showed that the African-American population had a lower REE than the Caucasian population. Some authors consider that race is essential when developing estimation formulas to improve the nutritional approach, especially in those that do not include body composition variables [55,56]. This omission could explain part of the lack of accuracy of some equations, since the ethnic groups present in the Chilean population, and their characteristics, are different from the populations in which the equations were generally developed (Europe, United States, etc.) [57,58]. In this sense, when population-specific equations are developed, it is possible to improve REE estimation [25,59]. For instance, in the adaptation carried out in our population, all the proposed formulas reduced the bias and the mean percentage difference. In addition, the classification percentage within ±10% was also increased. Wahrlich et al. [59] achieved a higher correct percentage of classification in women than we found in our results (77.5% vs. 51.4%) when they validated the formula proposed by Anjos et al. in a tropical urban population. However, their sample size was smaller, and the population characteristics were distinct.

In a study carried out in the Mexican population, the researchers developed an equation that improved REE estimation. However, no accuracy analysis was shown, so it is difficult to determine its real predictive capacity [60].

Cruz et al. [61], in a study of the Spanish population, achieved an R^2^ of 0.230 among those with a BMI of ≥ 25 kg/m^2^ using sex, age, and weight as independent variables. In our case, an R^2^ of 0.141 was reached in the group of 25 to <30 kg/m^2^. This rate reached 0.387 in the group of ≥30 kg/m^2^ with the same variables and larger sample size.

On the other hand, our results show less variability explained by the proposed formulas among women with normal weight and overweight. In these cases, it is possible that other sources of variation such as hormonal aspects, the performance of resistance training, or alterations that are not usually controlled in protocols, such as modifications of sleep patterns or quality, could alter the measurement of REE_IC_ [54,62,63].

From our perspective, the wide variety of proposed equations should not be a problem for its use. For instance, all of them could be incorporated into a mobile health application (mHealth) where values of the variables that any clinical professional manages in their particular clinical setting (with or without body composition analysis) could be introduced [64]. Finally, the app could use different equations—according to the data introduced—and show the estimated REEs, the formulas used, their accuracy, and the confidence margins for each one. The evidence clarifies that REE estimation is fundamental for adequate management of patients who need to regulate their energy balance through dietary interventions. Therefore, these tools would facilitate their implementation when the necessary equipment is not available due to high costs [65,66].

### Limitations and Strenghts

To our knowledge, there is not a study of this type in the Chilean population, so there is no possibility of comparing results. This research’s main limitations derive from the fact that it only considers healthy females without knowing their ethnicity.

Further, the range of the percentage of women within ±10% of REE_IC_ was between 47.7 and 58.6% depending on age and nutritional status. This percentage means that there is still a very high proportion (nearly half of the women) where their estimated REE is >10% different from their REE measured by indirect calorimetry. For this reason, it would be advisable to use calorimetry, if possible, to measure REE the first time a patient comes in for consultation. Subsequently, in following consultations (if a measurement is not possible), health professionals could use the equations, considering the estimation’s error. Nevertheless, the equations developed present a higher accuracy than those published in the literature, at least in healthy Chilean women.

Future studies should focus on some particular conditions seen in women that might influence energy expenditure, such as polycystic ovary syndrome, postpartum, or breast cancer [67,68,69]. Further, urinary nitrogen introduction would allow a more accurate measurement of resting energy expenditure through calorimetry. Finally, since this work has only focused on comparing equations in this specific population and showing that more accurate equations can be developed, the formulas must be validated in a different sample with similar characteristics but from the same population to ensure their accuracy.

## 5. Conclusions

The available equations for REE estimation are not accurate enough for application in Chilean adult women, regardless of age or nutritional status, so it is impossible to recommend and standardize its use. This study has shown that it is possible to develop more accurate equations adapted to this population’s characteristics, presenting greater accuracy than what is measured by indirect calorimetry. Finally, this research makes available different equations as alternatives to indirect calorimetry when it is impossible to perform it. However, only when and if the formulas are validated would it be possible to make use of them. The fact that the different formulas proposed are based on different variables also offers choosing one or another (being aware of each one’s estimation errors), depending on the tools or data to which health care professionals have access.

## Figures and Tables

**Table 1:** Estimation formulas for women.

Harris–Benedict * [31]		655.096 + (9.5634 × weight) + (1.849 × height) − (4.6756 × age)
Harris–Benedict modified by Roza * [26]		447.593 + (9.247 × weight) + (3.098 × height) − (4.330 × age)
Mifflin St Joer * [32]		(9.99 × weight) + (6.25 × height) − (4.92 × age) − 161
**Muller ^Ψ^** [33]		
**Weight**	BMI of > 18.5 to 25 kg/m^2^	(0.02219 × weight) + (0.02118 × height) − (0.01191 × age) + 1.233
	BMI of > 25 to 30 kg/m^2^	(0.04507 × weight) − (0.01553 × age) + 3.407
	BMI ≥ 30 kg/m^2^	(0.05 × weight) − (0.01586 × age) + 2.924
**FFM**	BMI of > 18.5 to 25 kg/m^2^	(0.0455 × FFM) + (0.0278 × FM) − (0.01291 × age) + 3.634
	BMI of > 25 to 30 kg/m^2^	(0.03776 × FFM) + (0.03013 × FM) − (0.01196 × age) + 3.928
	BMI ≥ 30 kg/m^2^	(0.05685 × FFM) + (0.04022 × FM) − (0.01402 × age) + 2.818
Owen * [34]		795 + (7.18 × weight)
Ireton-Jones * [35]		629 − (11 × age) + (25 × weight) − (609 × obesity) [obesity: present = 1; unpresented = 0]
Cunningham * [36]		500 + (22 × FFM)
Schofield * [37]		
18–30 years		14.818 × weight + 486.6
30–60 years		8.126 × weight + 845.6
≥60 years		9.082 × weight + 658.5
Katch–McArdle * [38]		370 + (21.6 × FFM)
**Henry and Rees ^Ψ^** [39]		
18–30 years		0.048 × weight + 2.562
30–60 years		0.048 × weight + 2.448
Oxford * [40]		
18–30 years		10.4 × weight + 615 × height (m) − 282
30–60 years		8.18 × weight + 502 × height (m) − 11.6
≥60 years		8.52 × weight + 421 × height (m) + 10.7

FM: fat mass; FFM: fat-free mass; BMI: body mass index. Unit of measurement: weight (kg); height (cm); FFM (kg), FM (kg); age (years); formulas with * (Kcal/day); formulas with ^Ψ^ (MJ/day).

**Table 2:** Characteristics of participants according to age groups and nutritional status.

			Age Groups			Nutritional Status Groups			
Variables	Total (*n* = 620)	18 to <30 Years (*n* = 128)	30 to <60 Years (*n* = 463)	≥60 Years (*n* = 29)	*p* ^ψ^	18.5 to <25 kg/m^2^ (*n* = 160)	25 to <30 kg/m^2^ (*n* = 254)	≥30 kg/m^2^ (*n* = 206)	*P* ^ω^
Age (years)	39.1 (11.4)	24.4 (3.3)	41.6 (8)	64.2 (3.7)	<0.001	36.4 (10.4)	40.2 (11.1) *	39.9 (12.1) *	<0.01
Weight (kg)	74 (14.3)	75.7 (17.8)	73.4 (13.3)	75.4 (12.7)	0.253	59.8 (7.4)	71.5 (6)	88.1 (13.1)	<0.001
Height (cm)	160.9 (6.3)	162.6 (7)	160.7 (6.1)	157.2 (5.2)	<0.001	161.7 (6.1) *	161.1 (6) *^‡^	160 (6.9) ^‡^	<0.05
BMI (kg/m^2^)	28.6 (5.2)	28.6 (6.6)	28.4 (4.8)	30.6 (5.6)	0.100	22.9 (2.6)	27.5 (1.4)	34.3 (3.9)	<0.001
BF%	37.9 (7.1)	36.4 (9.7) *	38 (6.2) *	41.7 (5.6)	<0.01	29.7 (6.1)	37.8 (3.4)	44.4 (3.7)	<0.001
FM (kg)	28.8 (10.4)	29 (13.2)	28.6 (9.6)	32 (9.3)	0.227	18.1 (5.7)	27.1 (4)	39.4 (8.7)	<0.001
FFM (kg)	45.1 (5.1)	46.6 (5.9)	44.8 (4.8) *	43.4 (4.1) *	<0.001	41.7 (3)	44.5 (3.8)	48.6 (5.6)	<0.001
BW (kg)	33 (3.6)	33.9 (3.8)	32.8 (3.6) *	31.8 (3) *	<0.01	30.5 (2.2)	32.4 (2.4)	35.6 (4.1)	<0.001

BMI: body mass index; BF%: body fat percentage; FM: fat mass; FFM: fat-free mass; BW: body water. *^‡^ Bonferroni test: non-significant difference between means with same symbol. ^ψ^ Bivariate analysis for age groups. ^ω^ Bivariate analysis for nutritional status groups.

**Table 3:** Resting energy expenditure (REE) (Kcal/day) by age groups.

Variables	Total (*n* = 620)	18 to <30 Years (*n* = 128)	30 to <60 Years (*n* = 463)	≥60 Years (*n* = 29)	*p*
**REE_IC_ (Kcal/kg/day)**	16.2 (2.9)	16.6 (3.2)	16.1 (2.8)	15.3 (2.6)	0.087
**REE_IC_**	1182.5 (252.9)	1227.6 (288.5)	1171.9 (241.1)	1153.6 (256.9)	0.072
**Harris–Benedict**	1476.3 (152.1)	1565.3 (173.1)	1459.8 (135.4)	1346.8 (123.4)	<0.001
Linear correlation	0.621 **	0.699 **	0.625 **	0.242	
Mean difference	293.8 (198.4)	337.7 (208.1)	287.9 (188.8)	193.2 (256.7)	
95% CI of mean difference	−95.1, 682.6	−70.2, 745.7	−82, 657.9	−309.9, 696.3	
% of difference	29.3 (25.9)	32.3 (26.5)	28.9 (25.7)	21.8 (25.9)	
*n* and % within ±10% of REE_IC_	107 (17.3)	16 (12.5)	85 (18.4)	6 (20.7)	
**Harris–Benedict modified by Roza**	1460 (150.2)	1545.4 (170.9)	1444.1 (134.3)	1334.6 (121.3)	<0.001
Linear correlation	0.621 **	0.702 **	0.624 **	0.231	
Mean difference	277.4 (198.3)	317.9 (207.9)	272.2 (189.5)	181 (257.5)	
95% CI of mean difference	−111.3, 666.1	−89.5, 725.3	−98.3, 642.8	−323.7, 685.7	
% of difference	27.9 (25.6)	30.7 (26)	27.5 (25.4)	20.7 (25.8)	
*n* and % within ±10% of REE_IC_	119 (19.2)	20 (15.6)	93 (20.1)	6 (20.7)	
**Mifflin St. Jeor**	1390.3 (173)	1491.3 (193.7)	1371.9 (155)	1238.1 (137.3)	<0.001
Linear correlation	0.615 **	0.701 **	0.618 **	0.211	
Mean difference	207.8 (200.3)	263.7 (205.8)	200 (189.7)	84.5 (264.6)	
95% CI of mean difference	−184.8, 600.3	−139.6, 667.1	−171.8, 571.8	−434, 603	
% of difference	21.5 (24.2)	25.8 (24.6)	20.9 (24)	11.9 (24.2)	
*n* and % within ±10% of REE_IC_	205 (33.1)	33 (25.8)	161 (34.8)	11 (37.9)	
**Owen**	1325.5 (102.9)	1338.4 (128.1)	1322.3 (95.6)	1321.3 (87.7)	0.285
Linear correlation	0.610 **	0.694 **	0.593 **	0.233	
Mean difference	143 (207)	110.8 (219.8)	150.4 (199.7)	167.7 (251.4)	
95% CI of mean difference	−262.6, 548.7	−320, 541.7	−241.1, 541.9	−325, 660.4	
% of difference	16.5 (24.1)	13.5 (23.1)	17.1 (24.2)	19.7 (26)	
*n* and % within ±10% of REE_IC_	243 (39.2)	56 (43.8)	181 (39.1)	6 (20.7)	
**Cunningham**	1493.2 (111.7)	1525.8 (129.7)	1486.6 (105.7) *	1455.3 (89.6) *	<0.001
Linear correlation	0.632 **	0.726 **	0.596 **	0.515 **	
Mean difference	310.7 (201.8)	298.2 (213.8)	314.7 (197.2)	301.7 (224.3)	
95% CI of mean difference	−84.9, 706.2	−120.7, 717.2	−71.9, 701.3	−137.9, 741.3	
% of difference	31.3 (27.1)	29.5 (26.2)	31.7 (27.1)	31.7 (30.1)	
*n* and % within ±10% of REE_IC_	90 (14.5)	20 (15.6)	67 (14.5)	3 (10.3)	
**Muller (Weight)**	1461.5 (159)	1539.7 (184.7)	1446.2 (144.8)	1360.3 (126)	<0.001
Linear correlation	0.630 **	0.706 **	0.622 **	0.286	
Mean difference	279 (196.4)	312.1 (205.1)	274.3 (188.7)	206, 6 (251.8)	
95% CI of mean difference	−106, 663.9	−89.8, 714.1	−95.6, 644.2	−286.8, 700.1	
% of difference	27.8 (25.5)	30 (25.5)	27.7 (25.5)	22.9 (25.8)	
*n* and % within ±10% of REE_IC_	118 (19)	21 (16.4)	92 (19.9)	5 (17.2)	
**Muller (Fat-free mass)**	1448.8 (143.8)	1516 (170.2)	1434.9 (131.4) *	1377.4 (112.2) *	<0.001
Linear correlation	0.647 **	0.717 **	0.625 **	0.624 **	
Mean difference	267.4 (193.2)	294.5 (202.1)	262.7 (189.4)	223.8 (206.4)	
95% CI of mean difference	−111.3, 646	−101.5, 690.6	−108.5, 633.8	−180.7, 628.3	
% of difference	27 (25.4)	28.7 (25.3)	26.8 (25.5)	24.1 (24.7)	
*n* and % within ±10% of REE_IC_	131 (21.2)	21 (16.7)	104 (22.5)	6 (20.7)	
**Ireton-Jones**	1843.4 (294.1)	2009.5 (281.1)	1817.6 (277.3)	1524.7 (209.2)	<0.001
Linear correlation	0.398 **	0.528 **	0.379 **	−0.088	
Mean difference	661 (302)	781.9 (276.8)	645.7 (290.5)	371.1 (345.3)	
95% CI of mean difference	69, 1253	239.3, 1324.5	76.4, 1215.1	−305.6, 1047.9	
% of difference	61.5 (37.5)	70 (35.9)	60.6 (37.5)	38.6 (35.1)	
*n* and % within ±10% of REE_IC_	25 (4)	1 (0.8)	21 (4.5)	3 (10.3)	
**Schofield**	1471 (170.7)	1608 (264.4)	1442.3 (108.2)	1324.2 (110.9)	<0.001
Linear correlation	0.565 **	0.694 **	0.593 **	0.233	
Mean difference	288.5 (210.4)	380.5 (217.4)	270.4 (197.1)	170.6 (255)	
95% CI of mean difference	−124, 701	−45.6, 806.6	−115.9, 656.8	−329.2, 670.4	
% of difference	28.9 (26.6)	35 (27)	27.7 (26.3)	19.8 (25.7)	
*n* and % within ±10% of REE_IC_	123 (19.8)	17 (13.3)	99 (21.4)	7 (24.1)	
**Katch–McArdle**	1345.1 (109.6)	1377.1 (127.3)	1338.6 (103.7) *	1307.9 (88) *	<0.001
Linear correlation	0.632 **	0.726 **	0.596 **	0.515 **	
Mean difference	162.6 (202.3)	149.6 (214.7)	166.7 (197.6)	154.3 (224.6)	
95% CI of mean difference	−233.9, 559.2	−271.1, 570.3	−220.6, 554.1	−285.9, 594.6	
% of difference	18.2 (24.2)	16.8 (23.4)	18.5 (24.3)	18.4 (27.1)	
*n* and % within ±10% of REE_IC_	245 (39.5)	50 (39.1)	187 (40.4)	8 (27.6)	
**Henry and Rees**	1439.1 (166.7)	1480.6 (204.7)	1427.6 (152.8)	-	<0.001
Linear correlation	0.627 **	0.694 **	0.593 **	-	
Mean difference	255.1 (197.1)	253 (207.7)	255.7 (194.3)	-	
95% CI of mean difference	−131.2, 641.4	−154.1, 660.2	−125.1, 636.5	-	
% of difference	25.7 (25.5)	24.7 (24.8)	26 (25.7)	-	
*n* and % within ±10% of REE_IC_	146 (24.7)	32 (25)	114 (24.6)	-	
**Oxford**	1413.6 (150.9)	1505.3 (201.9)	1395.6 (122.6)	1297.1 (110.5)	<0.001
Linear correlation	0.602 **	0.704 **	0.596 **	0.199	
Mean difference	231.1 (201.9)	277.8 (205)	223.7 (194.7)	143.5 (258.6)	
95% CI of mean difference	−164.7, 626.9	−124, 679.5	−157.9, 605.2	−363.4, 650.4	
% of difference	23.8 (25.1)	26.8 (24.7)	23.4 (25.1)	17.4 (25.5)	
*n* and % within ±10% of REE_IC_	167 (26.9)	29 (22.7)	131 (28.3)	7 (24.1)	

REE_IC_: resting energy expenditure measured by indirect calorimetry; CI: confidence interval; * non-significant difference between means with the same symbol. Linear correlation: Pearson’s correlation between REE measured by indirect calorimetry and REE estimated by equations; ** *p* < 0.001; without symbol: non-significant; Mean difference: paired t-test between REE estimated by equations and REE measured by indirect calorimetry; % of difference: (|REE−REEIC|REEIC×100); *n* and % within ±10% of REE_IC_: number and percentage of women with REE estimated by each formula within ±10% of REE_IC_; mean difference and 95% CI of mean difference are shown in Kcal/day; mean difference not statistically significant; all mean differences without a symbol are statistically significant (*p* < 0.01).

**Table 4:** Resting energy expenditure (Kcal/day) by nutritional status

Variables	18.5 to <25 kg/m^2^ (*n* = 160)	25 to <30 kg/m^2^ (*n* = 254)	≥30 kg/m^2^ (*n* = 206)	*p*
**REE_IC_ (Kcal/kg/day)**	17.7 (3)	16 (2.6)	15.1 (2.6)	<0.001
**REE_IC_**	1054.4 (185.2)	1142.8 (198)	1331.1 (285)	<0.001
**Harris–Benedict**	1352.1 (78.3)	1448.7 (91.7)	1606.8 (157)	<0.001
Linear correlation	0.259 **	0.359 **	0.623 **	
Mean difference	297.7 (181.5)	306 (185.9)	275.7 (223.9)	
95% CI of mean difference	−58, 653.4	−58.5, 670.4	−163, 714.4	
% of difference	32.1 (23.8)	30.7 (25.3)	25.5 (28)	
*n* and % within ±10% of REE_IC_	15 (9.4)	42 (16.5)	50 (24.3)	
**Harris–Benedict modified by Roza**	1340.3 (80.7)	1433.7 (93.6)	1585.1 (156.5)	<0.001
Linear correlation	0.272 **	0.364 **	0.624 **	
Mean difference	285.9 (180.8)	291 (185.6)	254 (223.7)	
95% CI of mean difference	−68.5, 640.3	−72.8, 654.7	−184.5, 692.5	
% of difference	30.9 (23.4)	29.3 (25)	23.8 (27.6)	
*n* and % within ±10% of REE_IC_	17 (10.6)	43 (16.9)	59 (28.6)	
**Mifflin St. Jeor**	1264 (102.6)	1362.4 (117.4)	1522.8 (184.8)	<0.001
Linear correlation	0.286 **	0.365 **	0.621 **	
Mean difference	209.6 (184.3)	219.6 (189.7)	191.7 (223.5)	
95% CI of mean difference	−151.7, 570.9	−152.2, 591.5	−245.2, 629.7	
% of difference	23.2 (21.9)	22.7 (23.9)	18.6 (26.2)	
*n* and % within ±10% of REE_IC_	41 (25.6)	82 (32.3)	82 (39.8)	
**Owen**	1221.7 (46)	1308.2 (43.1)	1427.5 (93.8)	<0.001
Linear correlation	0.242 **	0.380 **	0.592 **	
Mean difference	167.4 (179.7)	165.5 (185.9)	96.4 (241.6)	
95% CI of mean difference	−184.9, 519.6	−198.9, 529.8	−377.1, 569.9	
% of difference	19.5 (22.3)	18.2 (23.2)	12 (25.9)	
*n* and % within ±10% of REE_IC_	54 (33.8)	93 (36.6)	96 (46.6)	
**Cunningham**	1417.9 (66.6)	1478.3 (83.6)	1570.2 (122.2)	<0.001
Linear correlation	0.389 **	0.395 **	0.642 **	
Mean difference	363.5 (170.7)	335.5 (181.9)	239.1 (226.8)	
95% CI of mean difference	28.8, 698.1	−21.1, 692.1	−205.5, 683.6	
% of difference	38.5 (25.4)	33.4 (25.9)	23 (27.6)	
*n* and % within ±10% of REE_IC_	7 (4.4)	25 (9.8)	58 (28.2)	
**Muller (Weight)**	1324.6 (64.5)	1435.1 (82)	1600.4 (173)	<0.001
Linear correlation	0.293 **	0.372 **	0.621 **	
Mean difference	270.2 (177.4)	292.4 (184)	269.2 (223.4)	
95% CI of mean difference	−77.4, 617.9	−68.2, 653	−168.7, 707.1	
% of difference	29.4 (23.1)	29.5 (25)	24.9 (27.7)	
*n* and % within ±10% of REE_IC_	19 (11.9)	47 (18.5)	52 (25.2)	
Muller (Fat-free mass)	1330.2 (59.7)	1417.8 (61.2)	1579.3 (160.3)	<0.001
Linear correlation	0.446 **	0.336 **	0.635 **	
Mean difference	275.8 (167.4)	277.2 (183)	248.7 (221.6)	
95% CI of mean difference	−52.3, 603.8	−81.5, 636	−185.6, 683	
% of difference	29.9 (23.3)	28.2 (24.9)	23.4 (27.3)	
*n* and % within ±10% of REE_IC_	17 (10.6)	56 (22.2)	58 (28.3)	
**Ireton-Jones**	1714.1 (178) *	1974 (208.1)	1783.2 (381.3) *	<0.01
Linear correlation	0.243 **	0.358 **	0.621 **	
Mean difference	668.8 (223.6)	831.2 (230.1)	452.1 (302.7)	
95% CI of mean difference	221.6, 1098	380.2, 1282.2	−141.1, 1045.3	
% of difference	67.1 (31)	77.6 (35.5)	37.4 (32)	
*n* and % within ±10% of REE_IC_	2 (1.3)	0 (0)	23 (11.2)	
**Schofield**	1329.3 (74.5)	1439.1 (84)	1620.5 (188.7)	<0.001
Linear correlation	0.230 **	0.282 **	0.510 **	
Mean difference	274.9 (183.1)	296.4 (192)	289.4 (249)	
95% CI of mean difference	−83.9, 633.7	−80, 672.7	−198.6, 777.3	
% of difference	29.9 (23.9)	30 (25.8)	26.7 (29.5)	
*n* and % within ±10% of REE_IC_	20 (12.5)	49 (19.3)	54 (26.2)	
**Katch–McArdle**	1271.2 (65.4)	1330.5 (82)	1420.7 (120)	<0.001
Linear correlation	0.389 **	0.395 **	0.642 **	
Mean difference	216.8 (170.8)	187.7 (181.9)	89.6 (227.4)	
95% CI of mean difference	−117.9, 551.5	−168.8, 544.2	−356.1, 535.3	
% of difference	24.2 (22.8)	20 (23.3)	11.2 (24.8)	
*n* and % within ±10% of REE_IC_	41 (25.6)	94 (37)	110 (53.4)	
**Henry and Rees**	1274.6 (71.1)	1410.5 (71.3)	1605.1 (154.4)	<0.001
Linear correlation	0.351 **	0.367 **	0.601 **	
Mean difference	222 (164)	264.4 (185.9)	270 (229.9)	
95% CI of mean difference	−99.5, 543.5	−99.9, 628.7	−180.6 (720.5)	
% of difference	24.4 (21.7)	27 (25.2)	25.2 (28.6)	
*n* and % within ±10% of REE_IC_	40 (25.8)	57 (23.7)	49 (25.1)	
**Oxford**	1293.2 (84.5)	1388.9 (90.7)	1537.6 (159.8)	<0.001
Linear correlation	0.292 **	0.345 **	0.580 **	
Mean difference	238.9 (179.7)	246.1 (187.1)	206.5 (232.2)	
95% CI of mean difference	−113.4, 591.1	−120.7, 612.9	−248.7, 661.7	
% of difference	26.2 (22.5)	25.3 (24.5)	20.2 (27.2)	
*n* and % within ±10% of REE_IC_	33 (20.6)	60 (23.6)	74 (35.9)	

REE_IC_: resting energy expenditure measured by indirect calorimetry; CI: confidence interval. * Non-significant difference between means with the same symbol. Linear correlation: Pearson´s correlation between REE measured by indirect calorimetry and REE estimated by equations; ** *p* < 0.001; Mean difference: paired *t*-test between REE estimated by equations and REE measured by indirect calorimetry; % of difference: (|REE−REEIC|REEIC×100); *n* and % within ±10% of REE_IC_: number and percentage of women with REE estimated by each formula within ±10% of REE_IC_; mean difference and 95% CI of mean difference are shown in Kcal/day; all mean differences are statistically significant (*p* < 0.01).

**Table 5:** Linear correlations between REE_IC_ (Kcal/d) and independent variables.

Variables	Age	Weight	Height	BMI	BF%	FM	FFM	BW
**Total**								
REE_IC_	−0.143 **	0.625 **	0.281 **	0.529 **	0.415 **	0.561 **	0.632 **	0.628 **
**18.5 to <25 kg/m^2^**								
REE_IC_	−0.039	0.417 **	0.310 **	0.265 **	0.210 **	0.334 **	0.389 **	0.393 **
**25 to <30 kg/m^2^**								
REE_IC_	−0.144 *	0.380 **	0.330 **	0.147 *	0.085	0.239 **	0.395 **	0.339 **
**≥30 kg/m^2^**								
REE_IC_	−0.326 **	0.592 **	0.420 **	0.436 **	0.175 *	0.481 **	0.642 **	0.642 **
**18 to <30 years**								
REE_IC_	0.089	0.694 **	0.311 **	0.595 **	0.525 **	0.638 **	0.726 **	0.736 **
**30 to <60 years**								
REE_IC_	−0.159 **	0.593 **	0.278 **	0.498 **	0.381 **	0.528 **	0.596 **	0.594 **
**≥60 years**								
REE_IC_	−0.211	0.650 **	−0.100	0.660 **	0.591 **	0.656 **	0.515 **	0.518 **

REE_IC_: resting energy expenditure measured by indirect calorimetry; BMI: body mass index; BF%: body fat percentage; FM: fat mass; FFM: fat-free mass; BW: body Water; * *p* < 0.05; ** *p* < 0.01.

**Table 6:** Proposed formulas for resting energy expenditure estimation.

Based on Age, Weight, and Height											
Group		β	Equations Components	*p* ^ψ^	β Standardized	R^2^ Adjusted	Standard Error of Estimation	*p* ^ω^	Mean Difference (95%CI)	% of Difference (SD)	% within ±10% of REE_IC_
Total		495.015	Constant	–	–	0.411	194.1	<0.001	0 (193.8) −379.8, 379.8	3.1 (20.6)	50.2
		−3.312	Age (years)	<0.001	−0.149						
		11.044	Weight (kg)	<0.001	0.627						
Age	18 to <30 years	378.163	Constant	–	–	0.477	208.5	<0.001	0 (207.7) −407.1, 407.1	3.1 (20.6)	47.7
		10.741	Weight (kg)	<0.001	0.694						
	30 to <60 years	−136.460	Constant	–	–	0.356	193.4	<0.001	0 (193) −378.3, 378.3	3.2 (20.9)	50.3
		10.206	Weight (kg)	<0.001	0.564						
		3.479	Height (cm)	<0.05	0.088						
	≥60 years	159.660	Constant	–	–	0.401	198.8	<0.001	0 (195.3) −382.7, 382.7	3 (19)	51.7
		13.182	Weight (kg)	<0.001	0.650						
Nutritional Status	18 to <25 kg/m^2^	462.849	Constant	–	–	0.195	166.2	<0.001	0 (165.2) −323.8, 323.8	2.7 (18.2)	51.9
		−3.308	Age (years)	<0.05	–0.185						
		11.904	Weight (kg)	<0.001	0.474						
	25 to <30 kg/m^2^	245.434	Constant	–	–	0.141	183.4	<0.001	0 (183.1) −358.8, 358.8	3 (20.1)	49.2
		12.553	Weight (kg)	<0.001	0.380						
	≥30 kg/m^2^	466.087	Constant	–	–	0.387	224.3	<0.001	0 (223.2) −437.5, 437.5	3.6 (22.8)	45.1
		−4.583	Age (years)	<0.01	– 0.195						
		11.896	Weight (kg)	<0.001	0.545						
**Based on Age, Fat Mass, and Fat-Free Mass**											
**Group**		**β**	**Equations Components**	***p***	**β** **Standardized**	**R^2^ Adjusted**	**Standard Error of Estimation**	***p***	**Mean Difference (95%CI)**	**% of Difference (SD)**	**% within ±10% of REE_IC_**
Total		95.215	Constant	–	–	0.429	190.6	<0.001	0 (190.1) −372.6, 372.6	3.1 (20.4)	51.4
		−2.172	Age (years)	<0.01	–0.098						
		6.430	FM (kg)	<0.001	0.266						
		21.866	FFM (kg)	<0.001	0.426						
Age	18 to <30 years	−429.459	Constant	–	–	0.524	199.1	<0.001	0 (198.3) −388.7, 388.7	2.9 (19.2)	54.7
		35.538	FFM (kg)	<0.001	0.726						
	30 to <60 years	13.446	Constant	–	–	0.379	190	<0.001	0 (189.6) −371.7, −371.7	3.2 (20.8)	51.3
		5.608	FM (kg)	<0.001	0.222						
		22.265	FFM (kg)	<0.001	0.444						
	≥60 years	−257.714	Constant	–	–	0.238	224.2	<0.01	0 (220.2) −431.6, 431.6	4 (24.5)	58.6
		32.501	FFM (kg)	<0.01	0.515						
Nutritional Status	18 to <25 kg/m^2^	145.198	Constant	–	–	0.181	167.7	<0.001	0 (166.6) −326.5, 326.5	2.8 (18.7)	51.9
		7.070	FM (kg)	<0.01	0.216						
		18.724	FFM (kg)	<0.001	0.306						
	25 to <30 kg/m^2^	143.325	Constant	–	–	0.131	181	<0.001	0 (180.3) −353.4, 353.4	3 (20)	51.6
		7.046	FM (kg)	<0.05	0.146						
		18.196	FFM (kg)	<0.001	0.300						
	≥30 kg/m^2^	−2.251	Constant	–	–	0.433	214.6	<0.001	0 (213.6) −418.5, 418.5	3.4 (22.1)	51.9
		−3.961	Age (years)	<0.01	–0.169						
		30.661	FFM (kg)	<0.001	0.597						

FM: fat mass; FFM: fat-free mass; % within ±10% of REE_IC_: number and percentage of women with REE estimated by each formula within ±10% of REE_IC_; ^ψ^ *p* value of component; ^ω^ *p* value for model.

## Data Availability

The data presented in this study are available on request to the corresponding author.

## Supplementary Materials

The following are available online at https://www.mdpi.com/2072-6643/13/2/345/s1, Figure S1: Bland–Altman graph for Harris-Benedict versus indirect calorimetry, Figure S2: Bland–Altman graph for Harris-Benedict (Roza) versus indirect calorimetry, Figure S3: Bland–Altman graph for Mifflin St Jeor versus indirect calorimetry, Figure S4: Bland–Altman graph for Owen versus indirect calorimetry, Figure S5: Bland–Altman graph for Müller (Weight) versus indirect calorimetry, Figure S6: Bland–Altman graph for Cunningham versus indirect calorimetry, Figure S7: Bland–Altman graph for Müller (Fat-free Mass) versus indirect calorimetry, Figure S8: Bland–Altman graph for Ireton-Jones versus indirect calorimetry, Figure S9: Bland–Altman graph for Schofield versus indirect calorimetry, Figure S10: Bland–Altman graph for Katch-McArdle versus indirect calorimetry, Figure S11: Bland–Altman graph for Henry & Rees versus indirect calorimetry, Figure S12: Bland–Altman graph for Oxford versus indirect calorimetry, Table S1: Size effect (Cohen´s d) of the means difference of independent variables according to age and nutritional status groups, Table S2: Size effect (Cohen´s d) of Resting Energy Expenditure (Kcal/day) means difference between age groups, Table S3: Size effect (Cohen´s d) of Resting Energy Expenditure (Kcal/day) means difference between nutritional status groups, Table S4: Size effect (Cohen´s d) of means difference between Resting Energy Expenditure (Kcal/day) estimated by equations and measured by indirect calorimetry according to age and nutritional status groups.
