# Supplementary material for: Accuracy of the Resting Energy Expenditure Estimation Equations for Healthy Women
Source: Nutrients. 2021 Jan 24;13(2):345. doi: 10.3390/nu13020345 (PMC7912292; doi:10.3390/nu13020345)
Supplement: Supplementary file 1 [file nutrients-13-00345-s001.zip › Supplementary Tables S1 to S4.docx]

| **Table S1.** Size effect (Cohen´s d) of the means difference of independent variables according to age and nutritional status groups | | | | | | |
| --- | --- | --- | --- | --- | --- | --- |
|  | **Age groups** | | | **Nutritional status groups** | | |
| **Variables** | **18 to <30 years**  **and 30 to <60 years** | **18 to <30 years**  **and ≥60 years** | **30 to <60 years**  **and ≥60 years** | **18.5 to <25 kg/m^2^**  **and 25 to <30 kg/m^2^** | **18.5 to <25 kg/m^2^**  **and ≥30 kg/m^2^** | **25 to <30 kg/m^2^**  **and ≥30 kg/m^2^** |
| Age (years) | 2.37 | 11.79 | 2.89 | 0.35 | 0.31 | 0.03 |
| Weight (kg) | 0.16 | 0.02 | 0.15 | 1.78 | 2.58 | 1.69 |
| Height (cm) | 0.30 | 0.80 | 0.58 | 0.01 | 0.26 | 0.17 |
| BMI (kg/m^2^) | 0.04 | 0.31 | 0.45 | 2.36 | 3.36 | 2.42 |
| BF% | 0.23 | 0.58 | 0.44 | 1.75 | 3 | 1.87 |
| FM (kg) | 0.04 | 0.24 | 0.35 | 1.90 | 2.83 | 1.88 |
| FFM (kg) | 0.36 | 0.57 | 0.29 | 0.80 | 1.48 | 0.87 |
| BW (kg) | 0.30 | 0.57 | 0.28 | 0.82 | 1.50 | 0.98 |
| BMI: Body mass index; BF%: Body fat percentage; FM: Fat mass; FFM: Fat-free mass; BW: Body water | | | | | | |

| **Table S2.** Size effect (Cohen´s d) of Resting Energy Expenditure (Kcal/day) means difference between age groups | | | |
| --- | --- | --- | --- |
| Variables | **18 to <30 years**  **and 30 to <60 years** | **18 to <30 years**  **and ≥60 years** | **30 to <60 years**  **and ≥60 years** |
| REE_IC_ (Kcal/kg/day) | 0.17 | 0.42 | 0.28 |
| REE_IC_ | 0.22 | 0.26 | 0.08 |
| Harris Benedict | 0.73 | 1.32 | 0.84 |
| Harris Benedict modified by Roza | 0.71 | 1.29 | 0.82 |
| Mifflin St. Jeor | 0.73 | 1.37 | 0.87 |
| Owen | 0.16 | 0.14 | 0.01 |
| Cunningham | 0.35 | 0.57 | 0.30 |
| Muller (Weight) | 0.61 | 1.02 | 0.60 |
| Muller (Fat-free Mass) | 0.58 | 0.86 | 0.44 |
| Ireton-Jones | 0.69 | 1.80 | 1.07 |
| Schofield | 1.06 | 1.16 | 1.10 |
| Katch-McArdle | 0.35 | 0.57 | 0.30 |
| Henry & Rees | 0.30 | - | - |
| Oxford | 0.76 | 1.10 | 0.81 |

| **Table S3.** Size effect (Cohen´s d) of Resting Energy Expenditure (Kcal/day) means difference between nutritional status groups | | | |
| --- | --- | --- | --- |
| Variables | **18.5 to <25 kg/m^2^**  **and 25 to <30 kg/m^2^** | **18.5 to <25 kg/m^2^**  **and ≥30 kg/m^2^** | **25 to <30 kg/m^2^**  **and ≥30 kg/m^2^** |
| REE_IC_ (Kcal/kg/day) | 0.62 | 0.93 | 0.35 |
| REE_IC_ | 0.45 | 1.12 | 0.78 |
| Harris Benedict | 1.11 | 1.98 | 1.26 |
| Harris Benedict modified by Roza | 1.05 | 1.90 | 1.12 |
| Mifflin St. Jeor | 0.88 | 1.68 | 1.06 |
| Owen | 1.95 | 2.68 | 1.69 |
| Cunningham | 0.78 | 1.50 | 0.89 |
| Muller (Weight) | 1.46 | 2.02 | 1.26 |
| Muller (Fat-free Mass) | 1.44 | 1.97 | 1.39 |
| Ireton-Jones | 1.32 | 0.22 | 0.64 |
| Schofield | 1.36 | 1.94 | 1.29 |
| Katch-McArdle | 0.78 | 1.50 | 0.89 |
| Henry & Rees | 1.91 | 2.64 | 1.68 |
| Oxford | 1.08 | 1.85 | 1.18 |

| **Table S4.** Size effect (Cohen´s d) of means difference between Resting Energy Expenditure (Kcal/day) estimated by equations and measured by indirect calorimetry according to age and nutritional status groups | | | | | | | | |
| --- | --- | --- | --- | --- | --- | --- | --- | --- |
|  |  | **Age groups** | | | | **Nutritional status groups** | | |
| **Variables** | **Total** | **18 to <30 years** | **30 to <60 years** | **≥ 60 years** | **18.5 to <25 kg/m^2^** | | **25 to <30 kg/m^2^** | **≥30 kg/m^2^** |
| Harris Benedict | 1.41 | 1.42 | 1.47 | 0.96 | 2.09 | | 1.98 | 1.20 |
| Harris Benedict modified by Roza | 1.33 | 1.34 | 1.39 | 0.90 | 2.00 | | 1.88 | 1.10 |
| Mifflin St. Jeor | 0.96 | 1.07 | 0.99 | 0.41 | 1.40 | | 1.35 | 0.80 |
| Owen | 0.74 | 0.50 | 0.82 | 0.87 | 1.24 | | 1.15 | 0.45 |
| Cunningham | 1.59 | 1.33 | 1.69 | 1.57 | 2.61 | | 2.21 | 1.09 |
| Muller (Weight) | 1.32 | 1.29 | 1.38 | 1.02 | 1.95 | | 1.93 | 1.14 |
| Muller (Fat-free Mass) | 1.29 | 1.22 | 1.35 | 1.13 | 2.00 | | 1.88 | 1.07 |
| Ireton-Jones | 2.41 | 2.75 | 2.49 | 1.58 | 3.63 | | 4.09 | 1.34 |
| Schofield | 1.34 | 1.37 | 1.46 | 0.86 | 1.95 | | 1.95 | 1.20 |
| Katch-McArdle | 0.83 | 0.67 | 0.90 | 0.80 | 1.56 | | 1.24 | 0.41 |
| Henry & Rees | 1.20 | 1.01 | 1.27 | - | 1.57 | | 1.80 | 1.20 |
| Oxford | 1.11 | 1.12 | 1.17 | 0.73 | 1.66 | | 1.60 | 0.89 |
