# Supplementary figures and images for: Accuracy of the Resting Energy Expenditure Estimation Equations for Healthy Women
Source: Nutrients. 2021 Jan 24;13(2):345. doi: 10.3390/nu13020345 (PMC7912292; doi:10.3390/nu13020345)

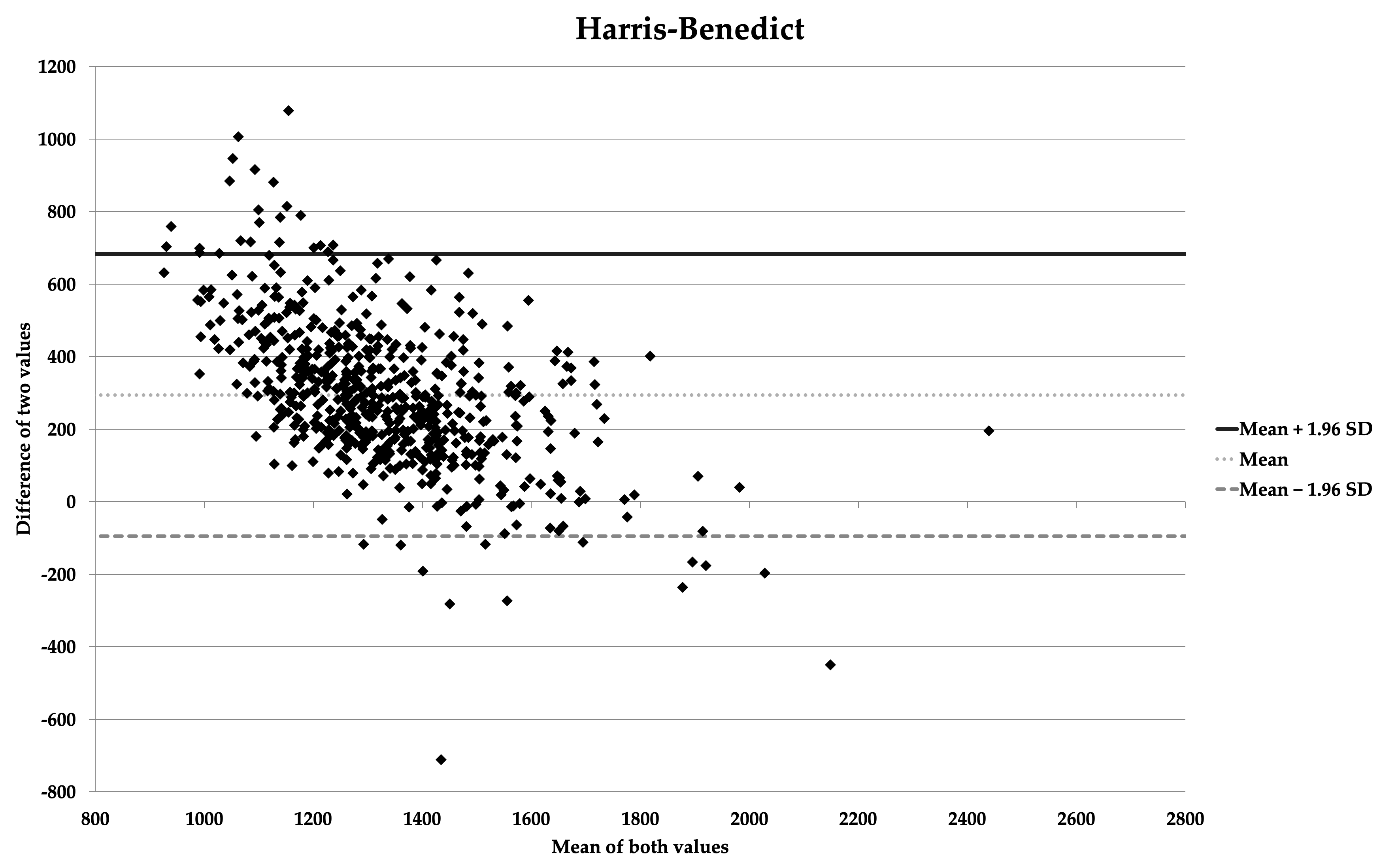

Supplement: Supplementary file 1 [file nutrients-13-00345-s001.zip › Figure S1. Harris-Benedict.png]

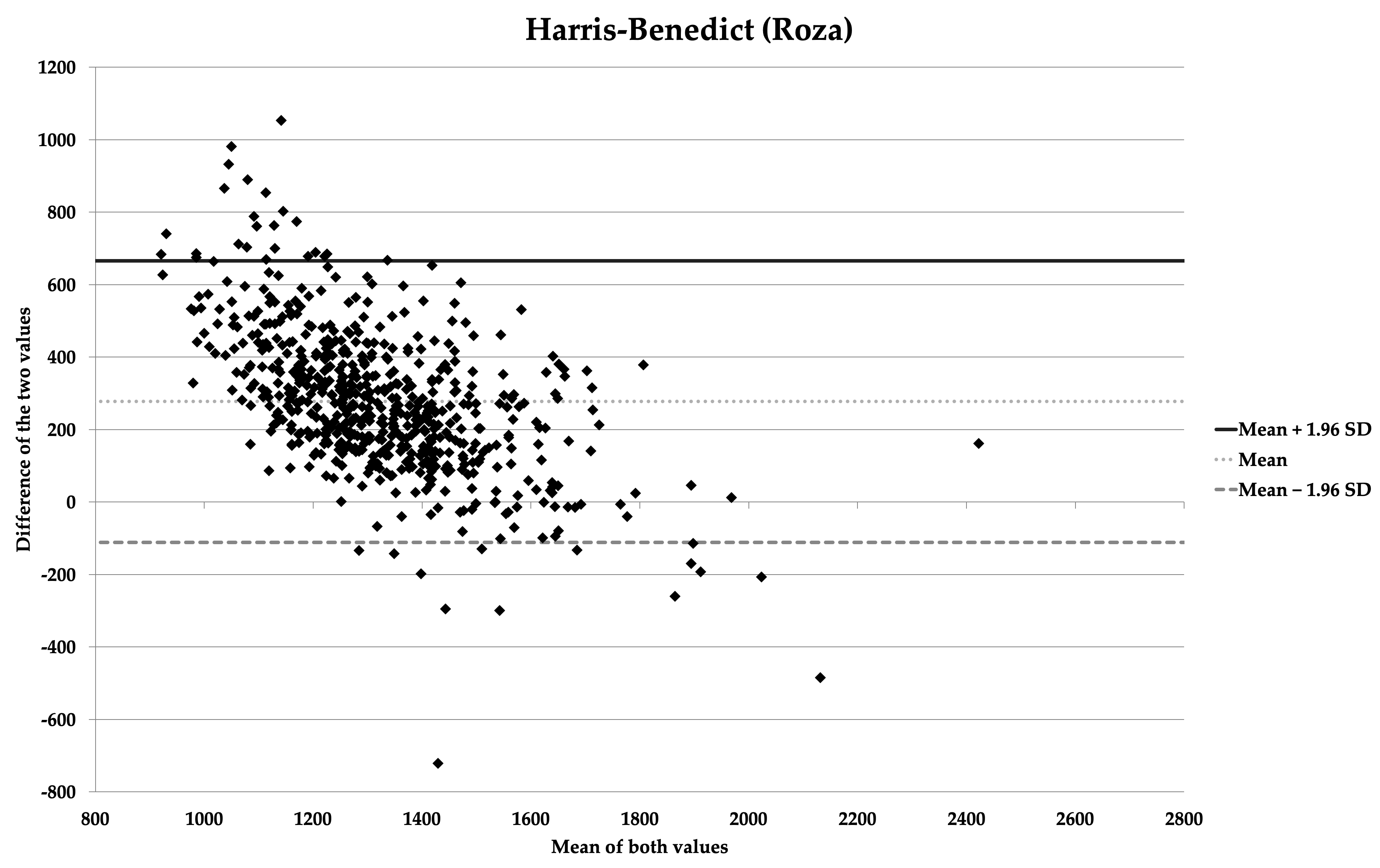

Supplement: Supplementary file 1 [file nutrients-13-00345-s001.zip › Figure S2. Harris-Benedict (Roza).png]

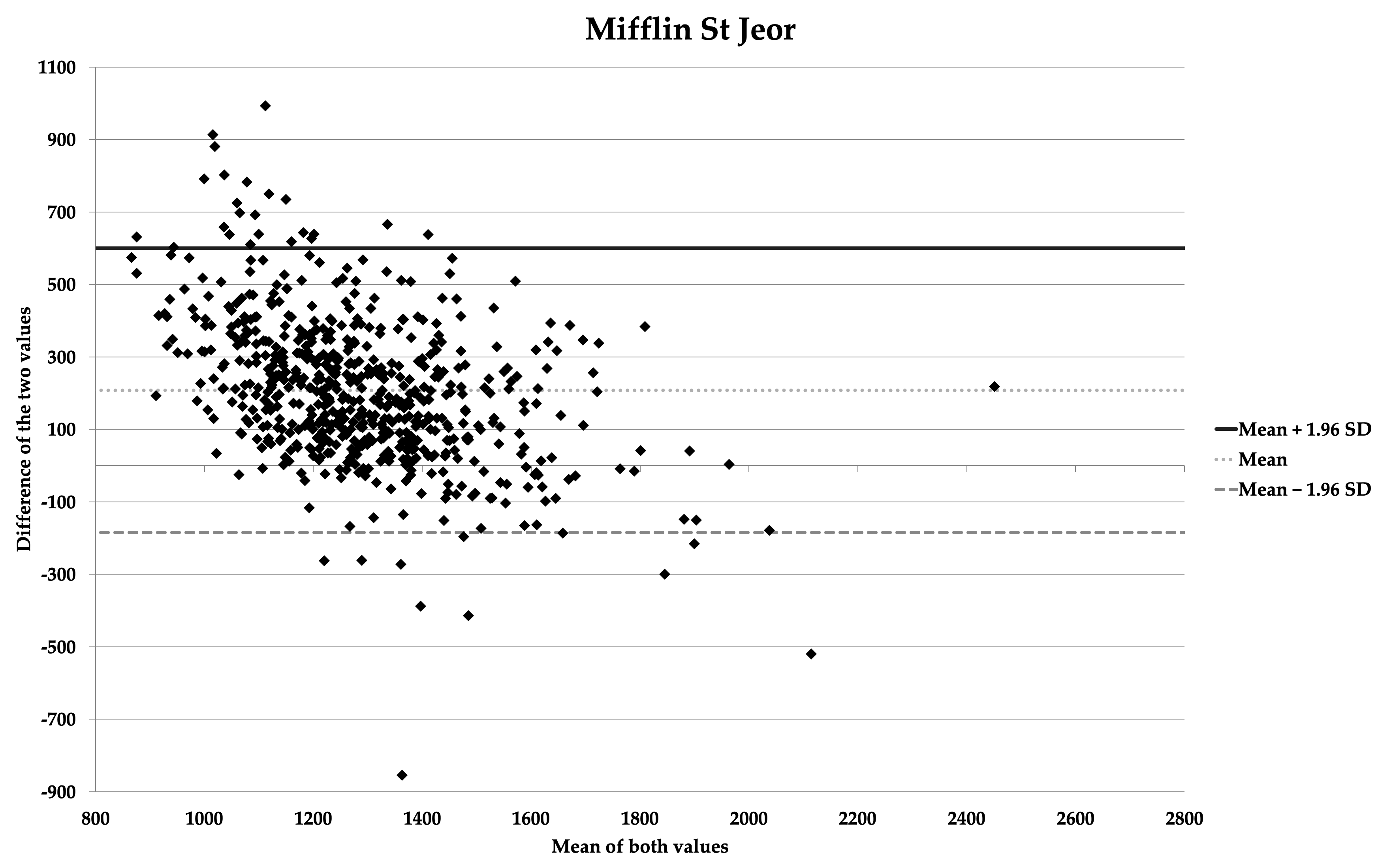

Supplement: Supplementary file 1 [file nutrients-13-00345-s001.zip › Figure S3. Mifflin St Jeor.png]

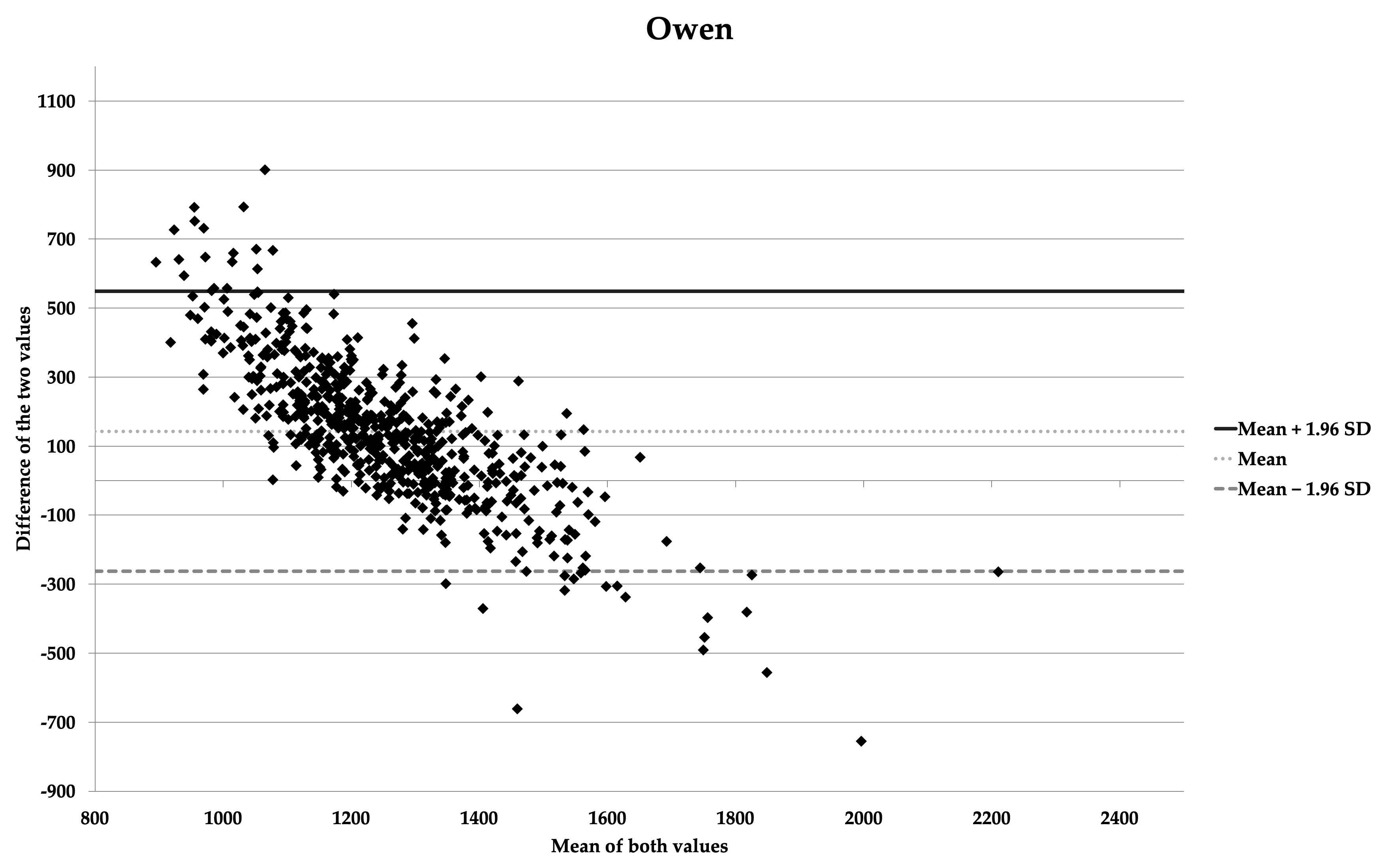

Supplement: Supplementary file 1 [file nutrients-13-00345-s001.zip › Figure S4. Owen.png]

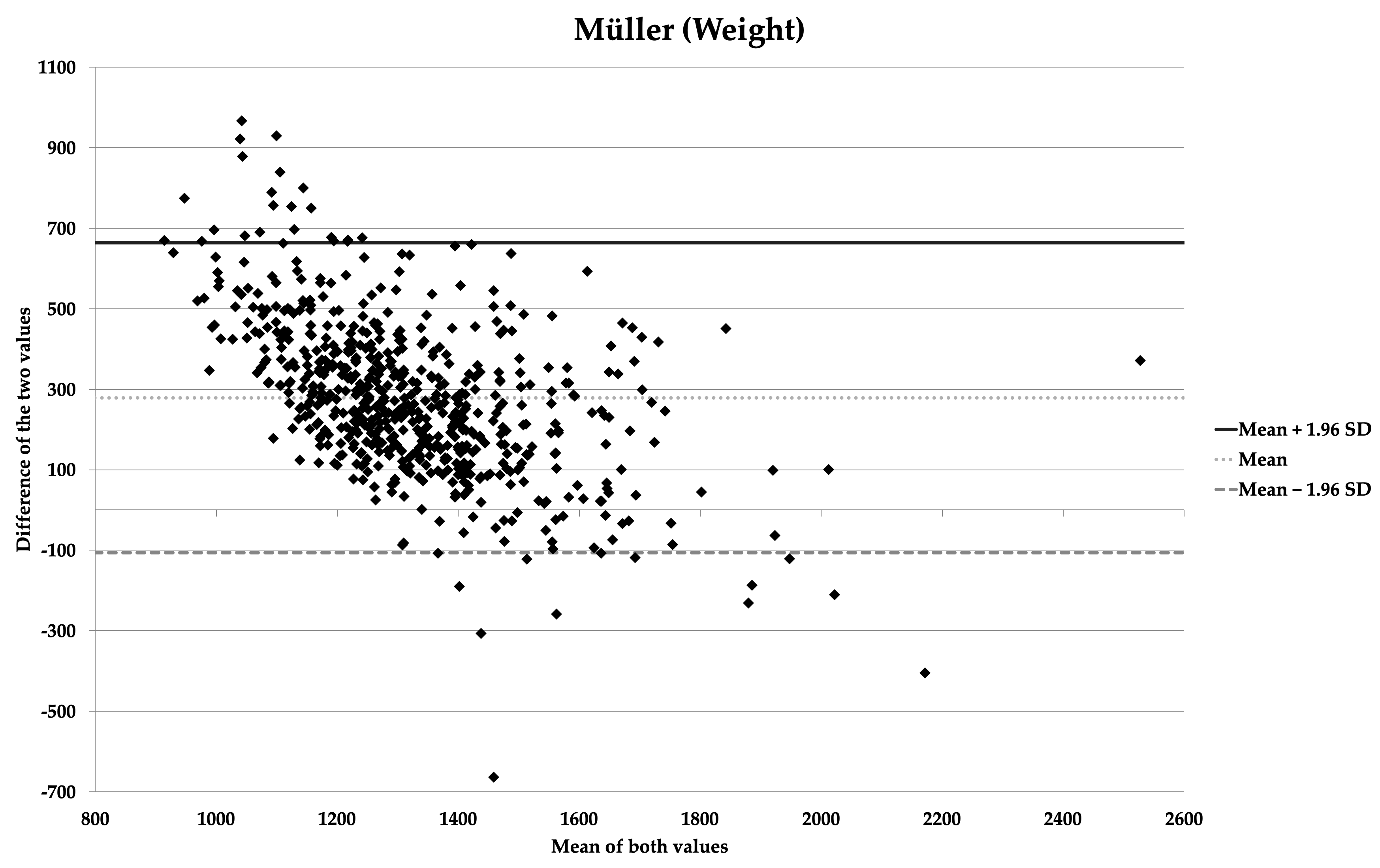

Supplement: Supplementary file 1 [file nutrients-13-00345-s001.zip › Figure S5. Mu╠êller (Weight).png]

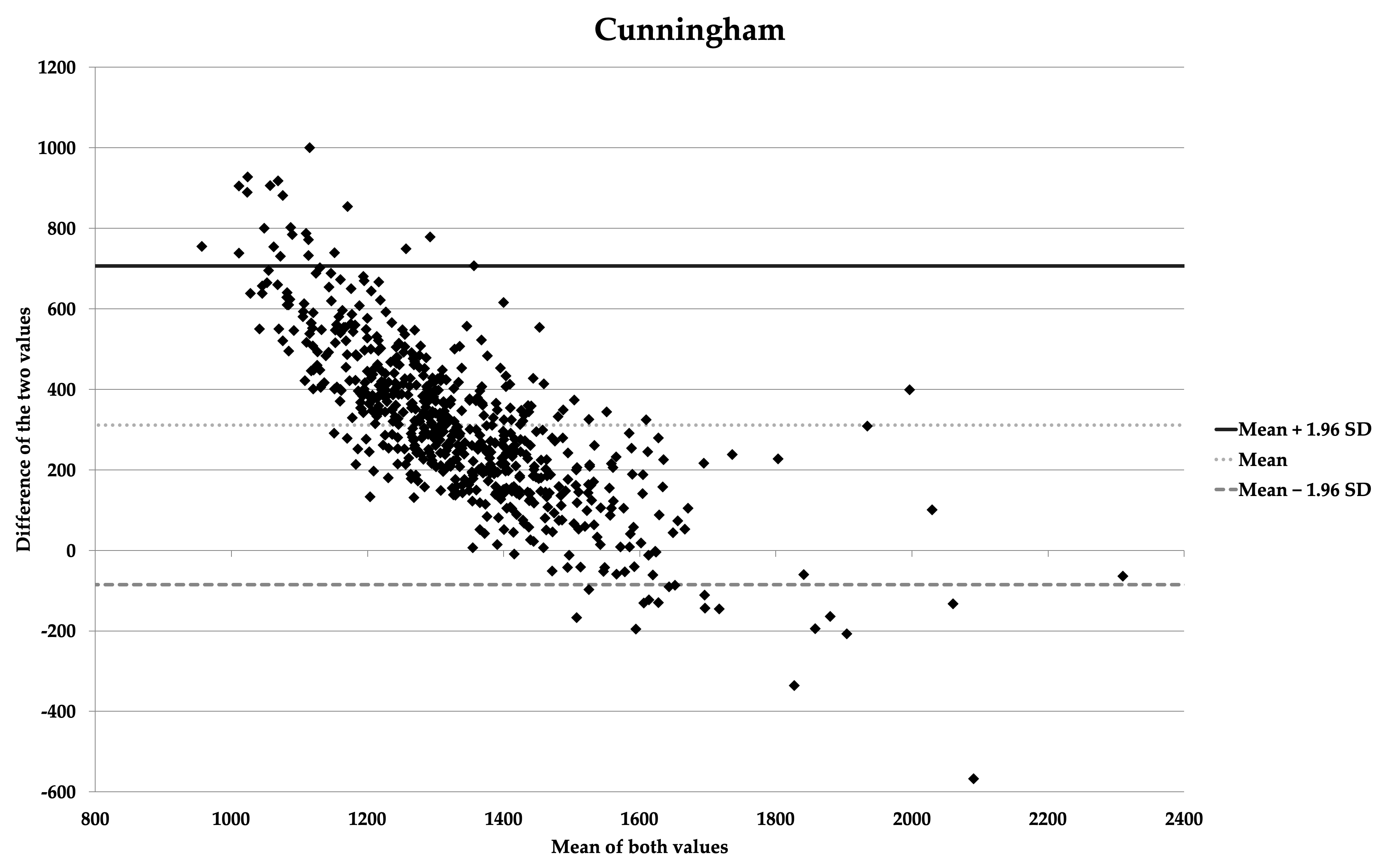

Supplement: Supplementary file 1 [file nutrients-13-00345-s001.zip › Figure S6. Cunningham.png]

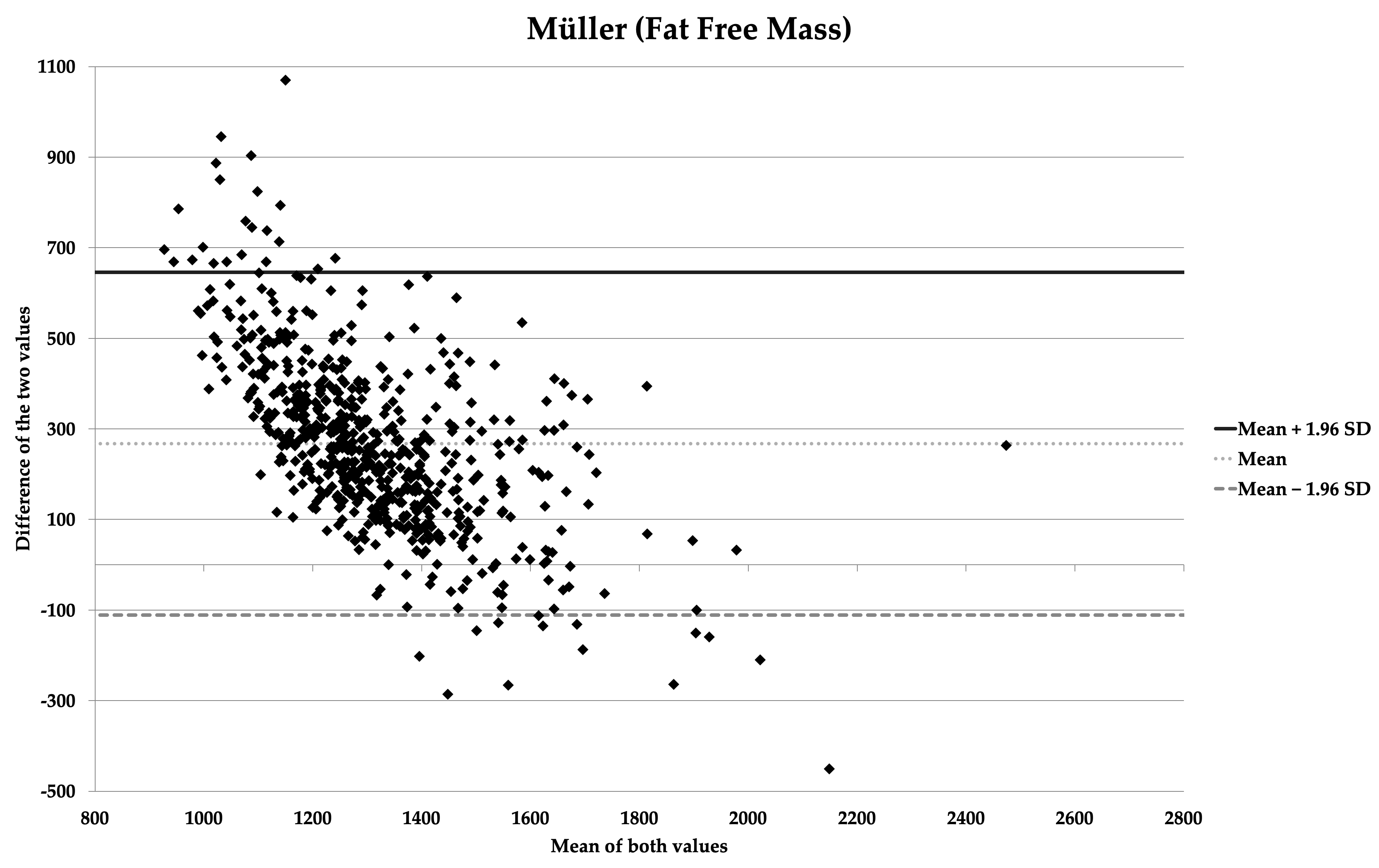

Supplement: Supplementary file 1 [file nutrients-13-00345-s001.zip › Figure S7. Muller (Fat-free Mass).png]

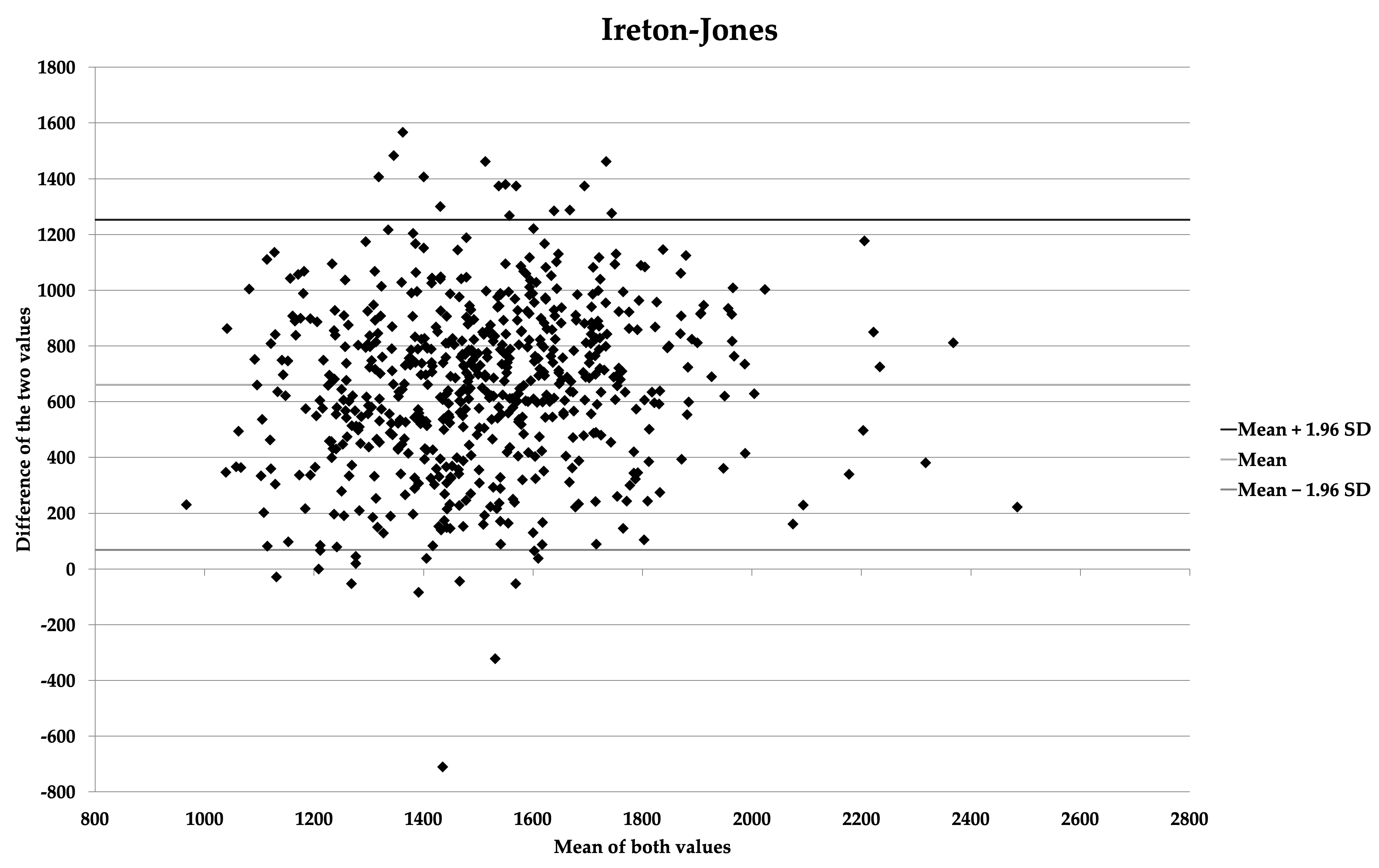

Supplement: Supplementary file 1 [file nutrients-13-00345-s001.zip › Figure S8. Ireton Jones.png]

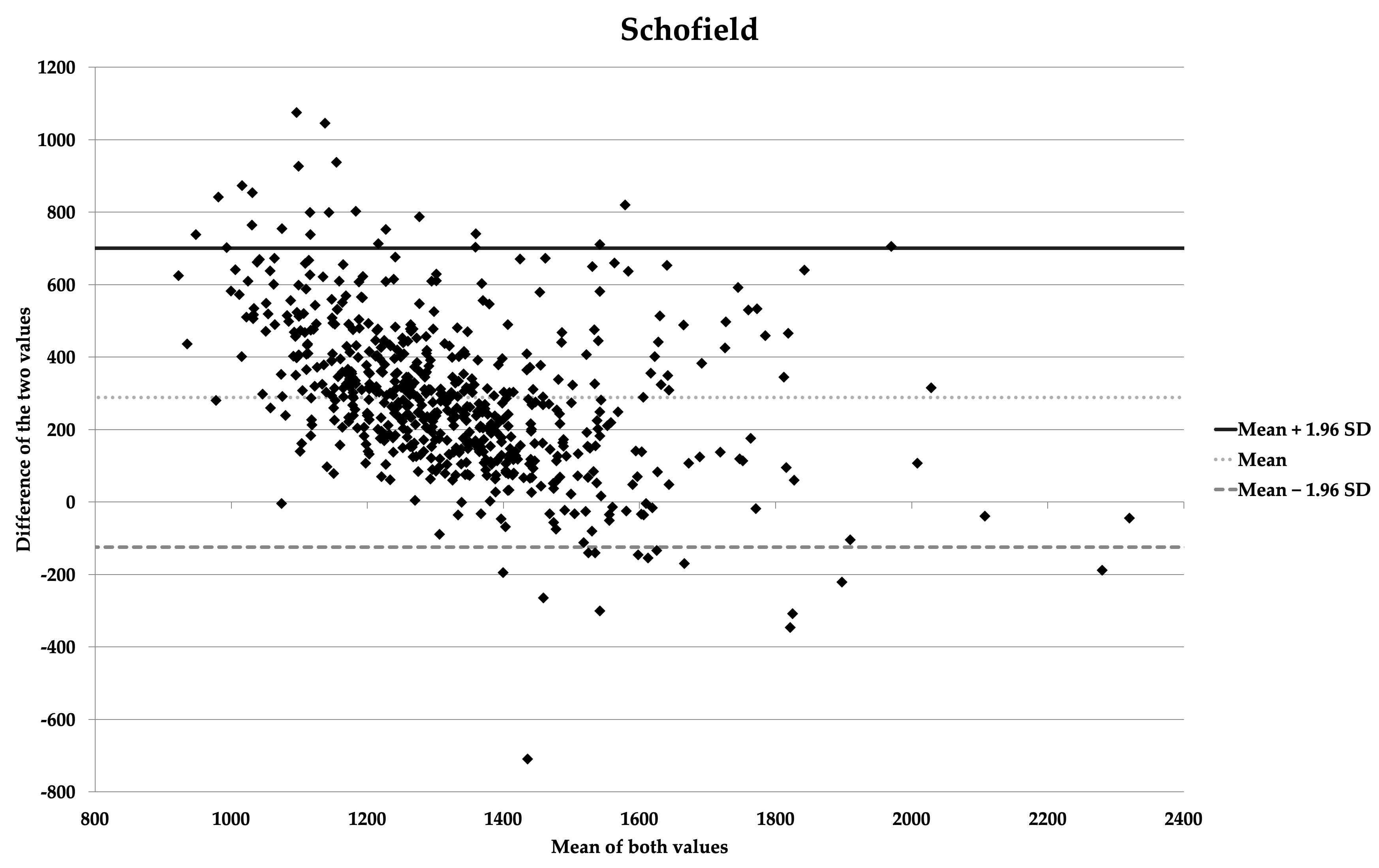

Supplement: Supplementary file 1 [file nutrients-13-00345-s001.zip › Figure S9. Schofield.png]

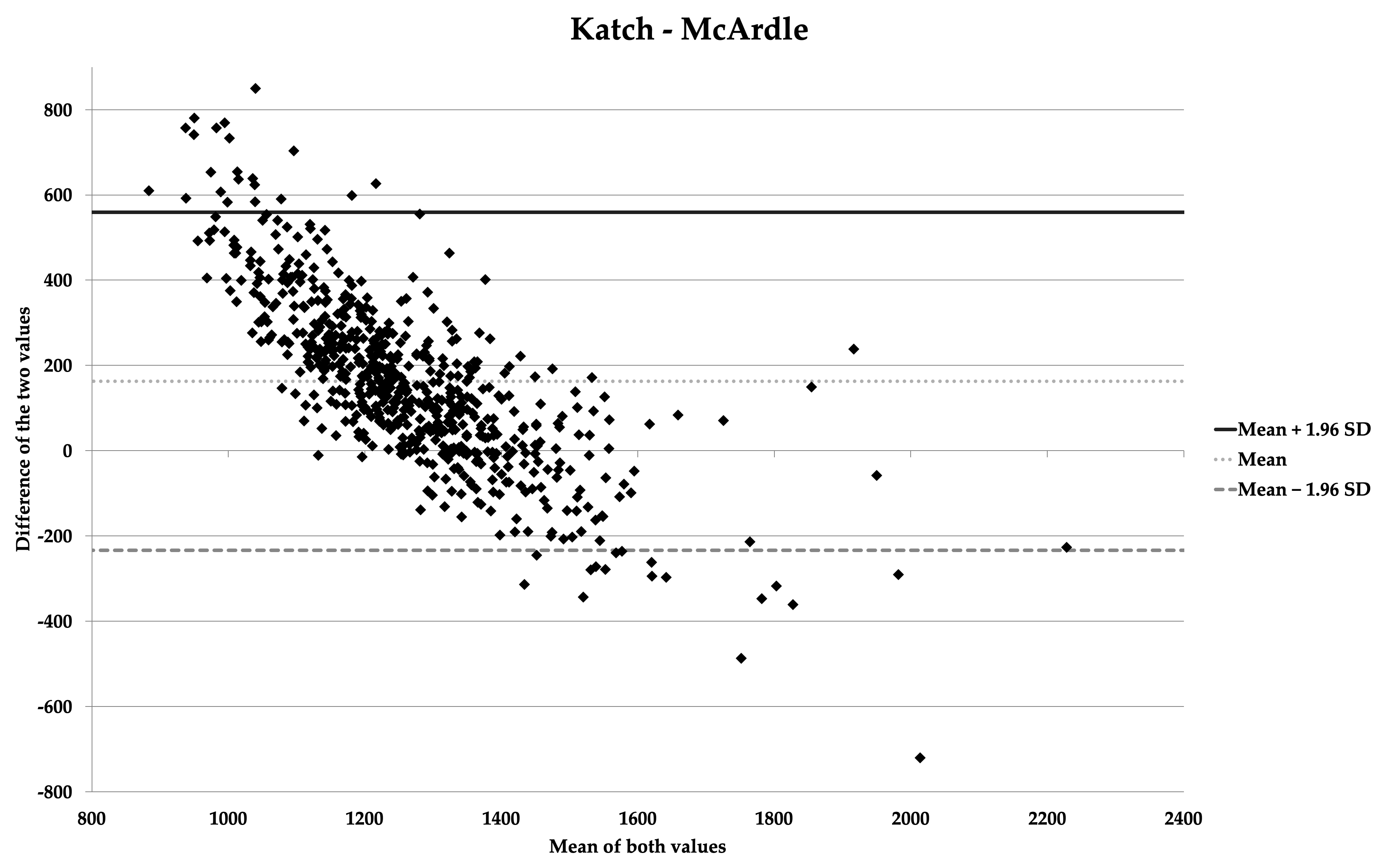

Supplement: Supplementary file 1 [file nutrients-13-00345-s001.zip › Figure S10. Katch - McArdle.png]

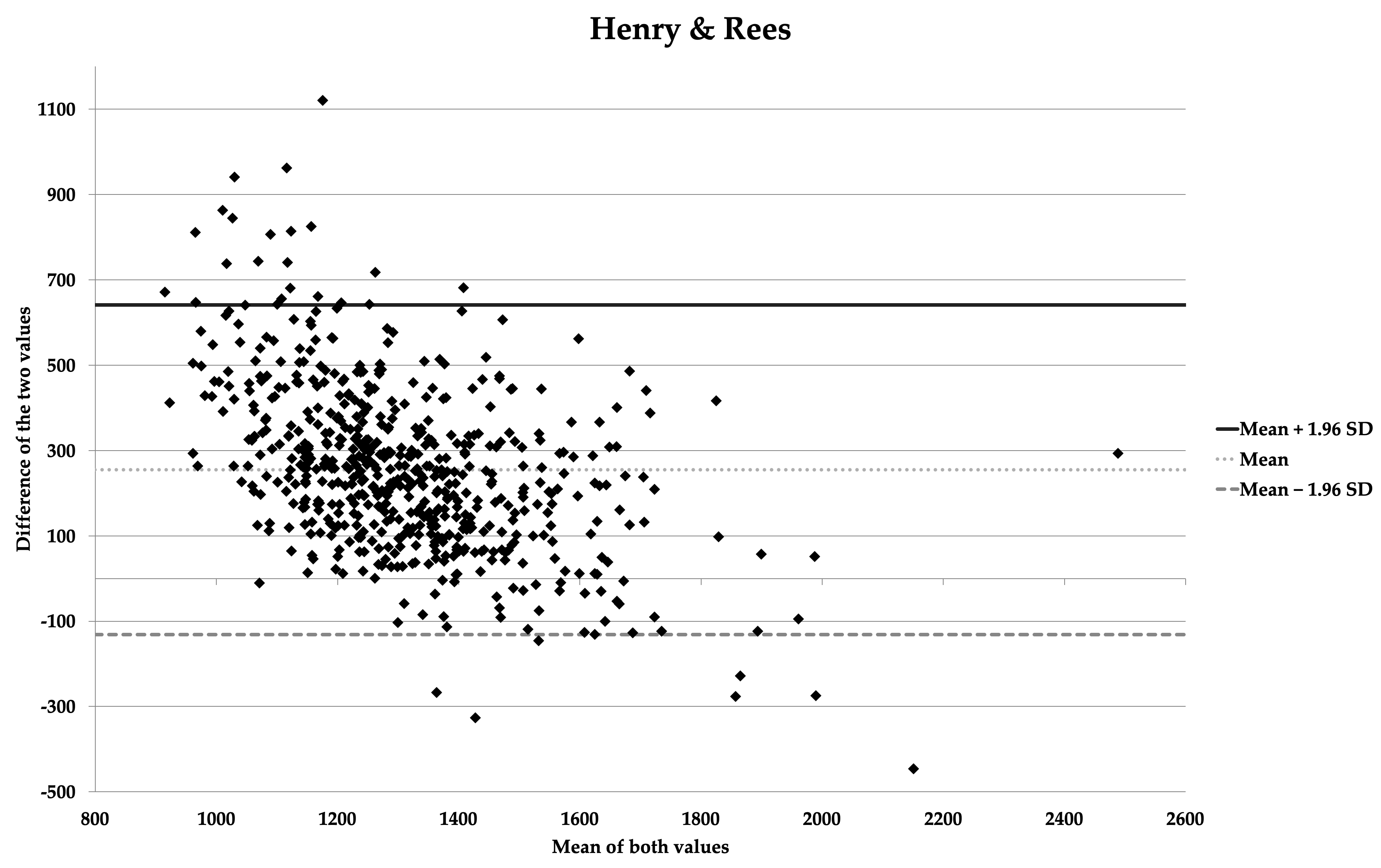

Supplement: Supplementary file 1 [file nutrients-13-00345-s001.zip › Figure S11. Henry & Rees.png]

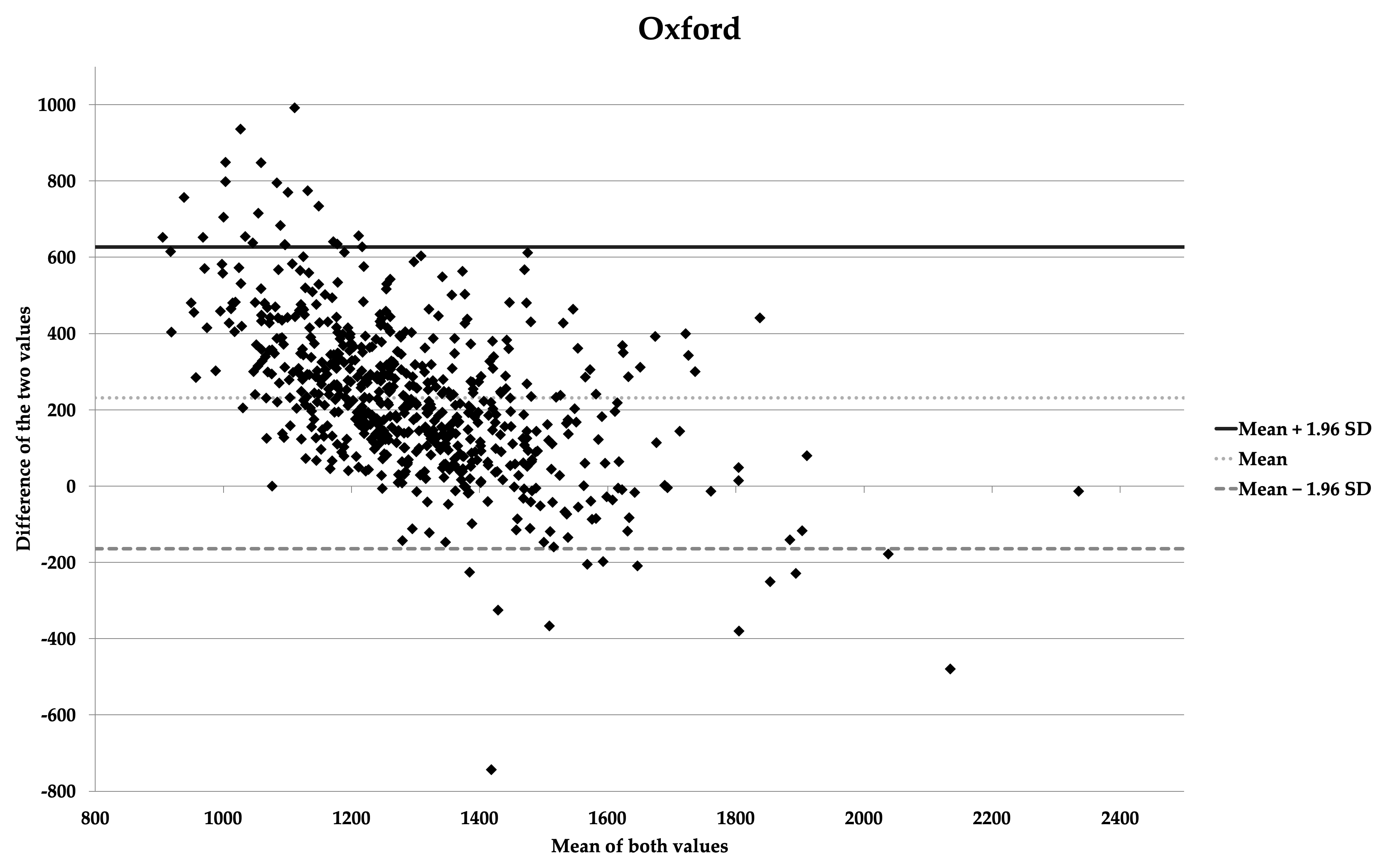

Supplement: Supplementary file 1 [file nutrients-13-00345-s001.zip › Figure S12. Oxford.png]
